# PclR is a transcriptional activator of the gene that encodes the pneumococcal collagen-like protein PclA

**DOI:** 10.1038/s41598-022-15758-7

**Published:** 2022-07-12

**Authors:** Ana Moreno-Blanco, Virtu Solano-Collado, Alejandro Ortuno-Camuñas, Manuel Espinosa, Sofía Ruiz-Cruz, Alicia Bravo

**Affiliations:** 1 Centro de Investigaciones Biológicas Margarita Salas, Consejo Superior de Investigaciones Científicas, Ramiro de Maeztu 9, 28040 Madrid, Spain; 2 Present Address: Institute of Medical Sciences, University of Aberdeen, Foresterhill, Aberdeen, AB25 2ZD UK; 3 Present Address: School of Microbiology, University College Cork and APC Microbiome Ireland, Western Road, Cork, T12 YT20 Ireland

**Keywords:** Microbiology, Molecular biology

## Abstract

The Gram-positive bacterium *Streptococcus pneumoniae* is a major human pathogen that shows high levels of genetic variability. The pneumococcal R6 genome harbours several gene clusters that are not present in all strains of the species. One of these clusters contains two divergent genes, *pclA*, which encodes a putative surface-exposed protein that contains large regions of collagen-like repeats, and *spr1404* (here named *pclR*). PclA was shown to mediate pneumococcal adherence to host cells in vitro. In this work, we demonstrate that PclR (494 amino acids) is a transcriptional activator. It stimulates transcription of the *pclA* gene by binding to a specific DNA site upstream of the core promoter. In addition, we show that PclR has common features with the Mga*Spn* transcriptional regulator (493 amino acids), which is also encoded by the R6 genome. These proteins have high sequence similarity (60.3%), share the same organization of predicted functional domains, and generate multimeric complexes on linear double-stranded DNAs. However, on the *PpclA* promoter region, Mga*Spn* binds to a site different from the one recognized by PclR. Our results indicate that PclR and Mga*Spn* have similar DNA-binding properties but different DNA-binding specificities, pointing to a different regulatory role of both proteins.

## Introduction

The core genome of a given bacterial species contains genes shared by all strains. In addition, the bacterial genomes often harbour a variable number of genes that are present in one or more, but not all, strains of the species. These accessory genes contribute to the high degree of genetic variability found in many bacterial species. The function of the accessory genes can be very diverse, including a wide range of adaptive traits that might be beneficial for the bacteria under certain environmental situations^1^. The Gram-positive bacterium *Streptococcus pneumoniae* (the pneumococcus) is a major human pathogen that shows high levels of genetic diversity. It is normally found as a harmless commensal of the upper respiratory tract (mainly the nasopharynx). Nevertheless, in individuals with a weakened immune system, the pneumococcus can migrate to other tissues/organs and cause life-threatening diseases, such as pneumonia, bacteraemia, and meningitis^2,3^. Despite the development of different vaccines and antibiotic therapies, *S. pneumoniae* remains a leading cause of morbidity and mortality worldwide, being the most common cause of bacterial pneumonia in children under five years old (https://www.who.int/en/news-room/fact-sheets/detail/pneumonia). An interesting aspect of *S. pneumoniae* is its capacity to incorporate exogenous DNA into its genome, which is mainly achieved by horizontal gene transfer mechanisms^4,5^ and plays an important role in its adaptation and evolution. Comparative genomic analyses have shown that over 20% of the coding sequences of any single pneumococcal isolate are not present in all strains^5–7^. Furthermore, it has been estimated that the rate at which the pneumococcus acquires genetic variation through recombination is much higher than the rate at which random mutations occur^8^.

The genome sequences of the pneumococcal strains TIGR4 (serotype 4) and R6 (a derivative of D39, serotype 2) were published in 2001^9,10^. A comparison of both sequences revealed that, among other differences, the R6 genome has six gene clusters that are absent from the TIGR4 genome^11^. One of the R6-specific clusters (9634 bp) consists of two divergent genes, *spr1403* (new locus tag: SPR_RS06970) and *spr1404* (new locus tag: SPR_RS06975). The *spr1403* gene encodes a putative cell wall anchored protein that contains large regions of collagen-like repeats, the number of which varies between strains^12^. This protein was named PclA for pneumococcal collagen-like protein A^12^. By using PCR techniques, Paterson *et al.*^12^ found that some clinical isolates from invasive pneumococcal disease harboured the *pclA-spr1404* gene cluster in the same genomic location as strain R6. Moreover, these authors showed that a *pclA* deletion mutant strain was defective in adhesion to host cells in vitro. The distribution of *pclA* was further analysed in a collection of pneumococcal clinical isolates from patients with community-acquired pneumonia^13^. This study showed that the presence of *pclA* was significantly associated with Pneumococcal Molecular Epidemiology Network (PMEN)^14^ clones, which suggested that PclA might contribute to the selection of prevalent clones^13^. The PMEN was established in 1997 to standardize the nomenclature and classification of antibiotic-resistant pneumococcal clones worldwide (https://www.pneumogen.net/pmen). Regarding the *spr1404* gene, it was reported that it encodes a putative transcriptional regulator^12^ but its function has not been investigated.

Global transcriptional regulators play crucial roles during bacterial adaptation to specific niches. They can rapidly adjust the gene expression pattern to new environmental conditions. The pneumococcal *mgaSpn* gene^15^, firstly named *mgrA*^16^, encodes a protein of 493 amino acids that is a member of the Mga/AtxA family of global transcriptional regulators^17^. This family includes Mga from *S. pyogenes*, AtxA from *Bacillus anthracis*, and MafR from *Enterococcus faecalis*^18–20^. Bioinformatics analyses have shown that Mga*Spn* is highly conserved in 25 pneumococcal strains whose genomes have been completely sequenced, including TIGR4 (locus *sp1800*) and R6 (locus *spr1622*)^15,21^. Signature-tagged mutagenesis in TIGR4 revealed that Mga*Spn* plays a significant role in both nasopharyngeal colonization and the development of pneumonia in murine infection models. Moreover, Mga*Spn* was shown to repress, directly or indirectly, the expression of several genes located within the *rlrA* pathogenicity islet^16,22^. This islet is absent from many pneumococcal strains^23^, including R6^10^. Further studies performed in the pneumococcal R6 strain demonstrated that Mga*Spn* functions as a transcriptional activator. It activates the transcription of a four-gene operon (*spr1623-spr1626*) by binding to a specific DNA site upstream of the *P1623B* promoter (positions − 60 to − 99)^15,17^. In vitro DNA binding experiments have shown that Mga*Spn* (i) generates multimeric complexes on linear double-stranded DNA fragments, (ii) binds to linear double-stranded DNAs with little or no sequence specificity, and (iii) has a preference for AT-rich DNA sites and for DNA regions that contain a potential intrinsic curvature. Because of these findings, we proposed that Mga*Spn* recognizes structural characteristics in its DNA targets rather than specific nucleotide sequences^17,24^.

In this work, we have investigated the function of the pneumococcal *spr1404* gene. We demonstrate that this gene (named *pclR* herein for *pclA* regulator) encodes a protein (PclR; 494 amino acids) that activates the transcription of the *pclA* gene in pneumococcal cells grown to mid-log phase under standard laboratory conditions. This activation requires a specific DNA site, which is located upstream of the *PpclA* core promoter. PclR interacts with such a site in vitro. Therefore, PclR could play a regulatory role in pneumococcal adhesion to human cells. In addition, we show that PclR and Mga*Spn* have high sequence similarity (60.3%), share the same organization of predicted functional domains, and display common features in their interaction with DNA. However, despite these similarities, these regulators have different DNA-binding specificities and different regulatory capacities.

## Results

### Organization of predicted functional domains in PclR

**Figure 1:**
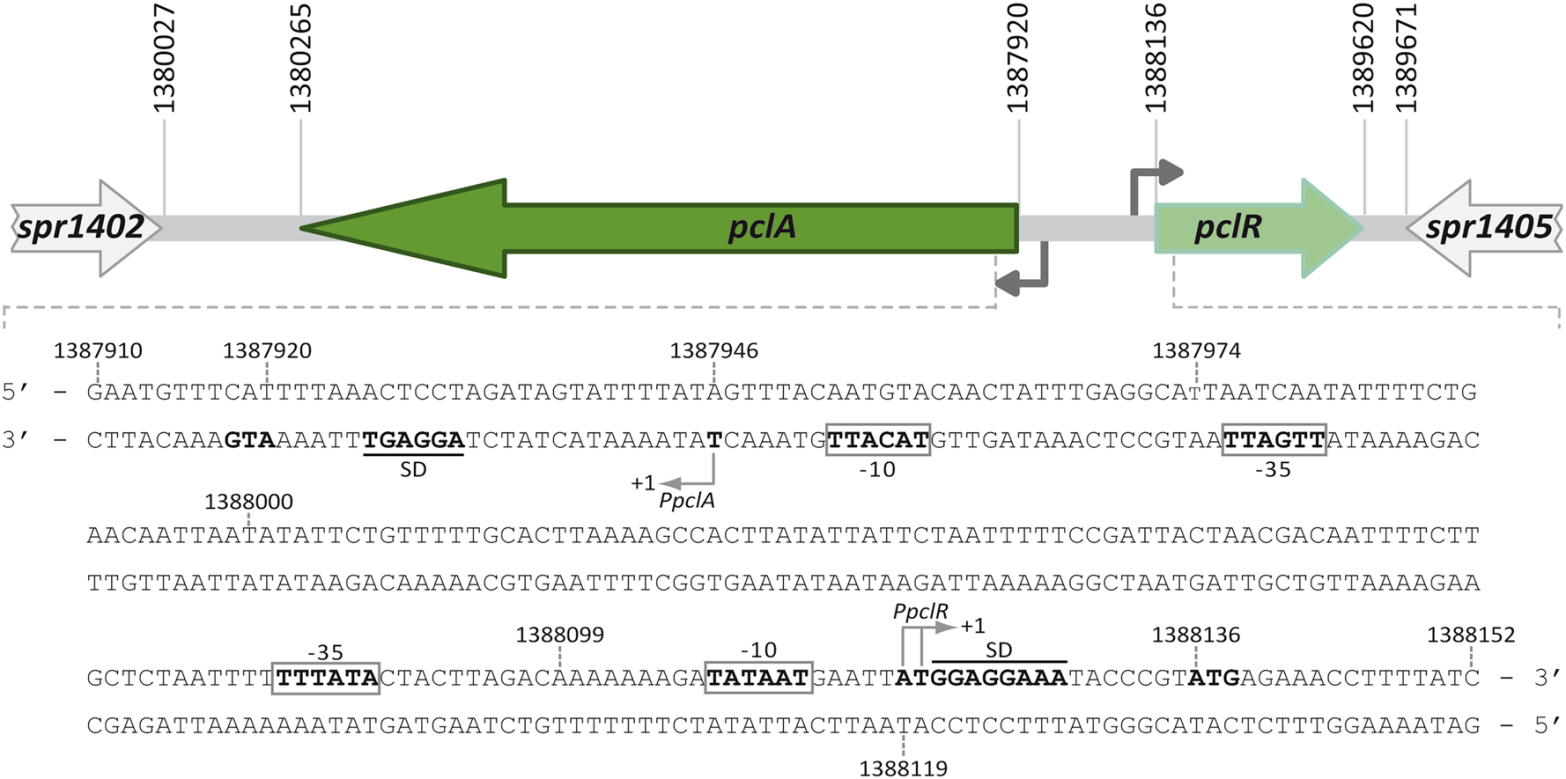
The R6-specific *pclA*-*pclR* cluster. Gene *spr1404* has been named *pclR* in this work. For each gene, the coordinates of the translation start and stop codons are indicated. Arrows upstream of the genes represent promoters. The nucleotide sequence of the region spanning coordinates 1,387,910 to 1,388,152 of the *S. pneumoniae* R6 chromosome is shown. The main sequence elements (− 35 box and − 10 box) of the promoters identified in this work (*PpclA* and *PpclR*) and the transcription start sites (+1 position) are indicated. The putative Shine-Dalgarno sequence (SD) and the translation start codon (ATG) of the *pclA* and *pclR* genes are indicated in boldface letters.

The pneumococcal R6 genome (NCBI RefSeq NC_003098.1)^10^ has several gene clusters that are absent from other pneumococcal genomes. One of them consists of the *spr1403* gene (*pclA*, pneumococcal collagen-like protein A)^12^ and the *spr1404* gene (here named *pclR*) (Fig. 1). The ATG codon at coordinate 1,388,136 is likely the translation initiation codon of the *pclR* gene, as it is preceded by a putative Shine-Dalgarno sequence (5′-GGAGGAAA-3′). Translation from this ATG codon results in a protein of 494 residues (PclR). EMBOSS Needle Pairwise Sequence Alignment^25,26^ of PclR and the pneumococcal Mga*Spn* transcriptional regulator (493 residues; locus_tag *spr1622*) revealed that these proteins have 60.3% of similarity and 40.1% of identity (Supplementary Fig. S1). According to the Conserved Domain Database (CDD)^27^ and the Protein Families Database (Pfam)^28^, PclR is predicted to have (i) two N-terminal helix-turn-helix DNA-binding domains, the so-called HTH_Mga (Family PF08280.14, residues 6 to 65) and Mga (Family PF05043.16, residues 72 to 158) domains, and (ii) a central phosphoenolpyruvate phosphotransferase system (PTS) regulation domain (PRD) (Family PRD_Mga PF08270.14, residues 174 to 391) (Supplementary Fig. S2). Moreover, the protein structure prediction server Phyre2^29^ revealed that the C-terminal region of PclR (residues 398 to 488) has structural homology to a PTS EIIB-like component. Thus, the organization of predicted functional domains in PclR is similar to the one reported for Mga*Spn*^15,24^. Supplementary Fig. S3 shows the predicted three-dimensional structure of the PclR monomer according to the AlphaFold Protein Structure Database (AlphaFold DB, https://alphafold.ebi.ac.uk)^30,31^, as well as the location of the predicted functional domains on such a structure. The AlphaFold Database predicts similar three-dimensional structures for the PclR and Mga*Spn* monomers (Supplementary Fig. S4).

### Expression of the *pclR* gene under laboratory conditions

By quantitative RT-PCR (qRT-PCR) assays and using the comparative *C*_T_ method^32^, we determined the relative expression of the *pclR* gene in pneumococcal R6 cells grown under standard laboratory conditions: AGCH medium supplemented with 0.2% yeast extract and 0.3% sucrose, 37 °C, and without aeration. Compared to the stationary phase, transcription of *pclR* was found to be higher (~ 3.2-fold) at the logarithmic growth phase (Supplementary Table S1). We also determined the relative expression of the regulatory *mgaSpn* gene. Like *pclR*, transcription of *mgaSpn* was higher (~ 4.3-fold) in exponentially growing R6 cells (Supplementary Table S2). Thus, all the experiments shown in this work were performed during the logarithmic growth phase.

Paterson *et al.* (2008) constructed a *pclR* (*spr1404)* deletion mutant strain by allelic replacement with a spectinomycin resistance cassette^12^. Using such a mutant, they found that the lack of *pclR* had no significant effect on the intracellular levels of *pclA* transcripts^12^. This finding suggested to us that, under laboratory conditions, an increase in the expression of the *pclR* gene could be necessary to detect an effect on the transcription of the *pclA* gene. Therefore, to test this hypothesis (see below), we constructed two R6 derivative strains designed to produce different levels of PclR. Specifically, we inserted the promoterless *pclR* gene into the pDLF constitutive expression vector^20^ in both orientations, generating the recombinant plasmids pDLF*pclR* (expression of *pclR*) and pDLF*pclR-i* (no expression of *pclR*). Then, we introduced each recombinant plasmid into the R6 strain. By qRT-PCR, we determined the relative expression of the *pclR* gene in both strains: R6/pDLF*pclR* (expression of *pclR* from the chromosome and the plasmid) and R6/pDLF*pclR-i* (expression of *pclR* only from the chromosome). As expected, the amount of *pclR* transcripts was higher (~ 3.1-fold) in strain R6/pDLF*pclR* (Supplementary Table S3). Each recombinant plasmid was also introduced into the R6∆*mga* mutant strain, which lacks the *mgaSpn* gene^15^. As shown in Supplementary Table S4, the amount of *pclR* transcripts was higher (~ 4.9-fold) in strain R6∆*mga*/pDLF*pclR* compared to strain R6∆*mga*/pDLF*pclR-i*. In the next sections, we will refer to R6/pDLF*pclR* and R6∆*mga*/pDLF*pclR* as strains with high levels of *pclR* expression, and to R6/pDLF*pclR-i* and R6∆*mga*/pDLF*pclR-i* as strains with low levels of *pclR* expression.

### Identification of the promoter of the *pclR* gene

**Figure 2:**
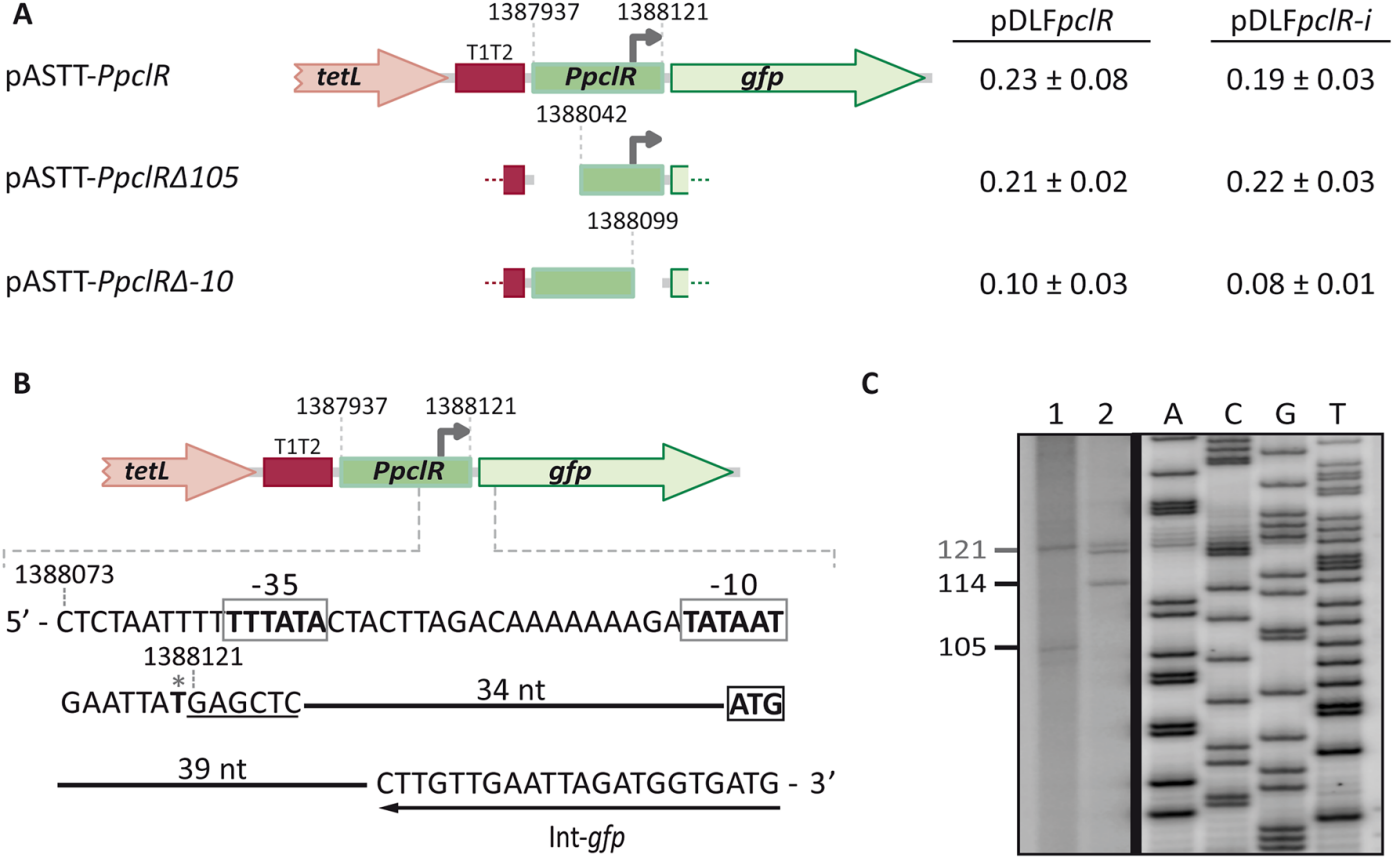
Identification of the *PpclR* promoter. **(A)** Fluorescence assays. Three regions of the R6 chromosome were amplified by PCR and inserted into the *Sac*I site of the promoter-probe vector pASTT. The coordinates of the inserted regions are indicated. The *tetL* gene confers resistance to tetracycline^33^. The promoter-less *gfp* gene encodes a variant of the green fluorescent protein^34^. The T1T2 box represents the tandem transcriptional terminators T1 and T2 of the *E. coli rrnB* ribosomal RNA operon^35^. The grey arrow represents the − 10 element of the *PpclR* promoter. Each pASTT derivative was introduced into strain R6∆*mga*/pDLF*pclR* (high levels of *pclR* expression) and strain R6∆*mga*/pDLF*pclR-i* (low levels of *pclR* expression). The intensity of fluorescence (arbitrary units) corresponds to 0.8 ml of culture (OD_650_ = 0.4). In each case, three independent cultures were analysed. **(B)** Plasmid pASTT-*PpclR*. The main sequence elements (− 35 box and − 10 box) of the *PpclR* promoter and the ATG translation start codon of the *gfp* gene are indicated. The *Sac*I site is underlined. The position of the Int-*gfp* oligonucleotide used for primer extension is shown. The asterisk indicates the transcription start site identified in this work. **(C)** Analysis of primer extension reactions. Lane 1: total RNA from R6/pASTT-*PpclR* cells and oligonucleotide Int-*gfp*. Lane 2: total RNA from R6 cells and oligonucleotide Dw*1404*-2. Dideoxy-mediated chain termination sequencing reactions (M13mp18 DNA and primer − 40 M13^36^) were run in the same gel as DNA size markers. The corresponding lanes (A, C, G, and T) came from the same gel but were taken at a lower exposure (delineation with dividing lines). See the full-length gel (high and low exposures) at the end of the Supplementary Information file. The size (in nucleotides) of the cDNA products is indicated on the left of the gel.

The BPROM program (*Softberry, Inc.*) predicts a promoter sequence (named *PpclR* herein) upstream of the *pclR* gene. It has a canonical − 10 element (**TATAAT**) and a possible − 35 element (**TT**T**A**T**A**) at the suboptimal spacer length of 19 nucleotides (Fig. 1). By transcriptional fusions, we analysed the promoter activity of such a sequence (Fig. 2A). A 185-bp DNA fragment (coordinates 1,387,937 to 1,388,121) was inserted into the pASTT promoter-probe vector, which is based on the *gfp* reporter gene. The recombinant plasmid (pASTT-*PpclR*) was then introduced into R6∆*mga*/pDLF*pclR* (high levels of *pclR* expression) and R6∆*mga*/pDLF*pclR-i* (low levels of *pclR* expression). In both strains, *gfp* expression was ~ 2.5-fold higher than the basal level (strains harbouring pASTT, 0.08 ± 0.02 units). Similar results were obtained with the plasmid pASTT-*PpclR∆105* (Fig. 2A). These results showed that (i) the 80-bp region between coordinates 1,388,042 and 1,388,121 contains a promoter sequence, and (ii) different levels of *pclR* expression do not affect the activity of such a promoter (no autoregulation). Furthermore, no promoter activity was detected when the region between coordinates 1,388,099 and 1,388,121 was deleted (plasmid pASTT-*PpclR∆-10*) (Fig. 2A). Such a deletion removes the − 10 element of the *PpclR* promoter (see Fig. 1).

The transcription start site of the *pclR* gene was identified by primer extension assays. We used total RNA from R6 cells and the oligonucleotide Dw*1404*-2 (coordinates 1,388,208 to 1,388,232) (Table 1). A cDNA product of 114 nucleotides was detected (Fig. 2C, lane 2), which could correspond to a transcription initiation event at coordinate 1,388,119. This coordinate is located 6 nucleotides downstream of the − 10 element of the *PpclR* promoter (Fig. 1). Additionally, we performed primer extension assays with total RNA from R6 cells harbouring pASTT-*PpclR*. In this plasmid, the *gfp* reporter gene is under the control of the *PpclR* promoter (Fig. 2A). As a primer, we used the oligonucleotide Int-*gfp* (Table 1), which anneals to *gfp* transcripts (Fig. 2B). A cDNA product of 105 nucleotides was detected (Fig. 2C, lane 1), which could correspond to a transcription initiation event at coordinate 1,388,120. This coordinate is located 7 nucleotides downstream of the − 10 element of the *PpclR* promoter (Fig. 1). In addition to the mentioned cDNA products, a possible non-specific product of 121 nucleotides was detected in both primer extension reactions (Fig. 2C, lanes 1 and 2). From these results, we conclude that the pneumococcal RNA polymerase recognizes the *PpclR* promoter and initiates transcription at coordinate 1,388,119/1,388,120 (Fig. 1).

**Table 1:** Oligonucleotides used in this work.

Name	Sequence (5′ to 3′)^a^
*FpclR*	CTTAGACAAAAAA**GC**AT**GC**AATGAATTATGG
*RpclR*	GTAAAGGAAGTATAG**C**A**TG**CAGATAAGAGAA
Dw*pclA*	CATTTTAAACTCC**G**AG**C**T**C**GTATTTTA
*pclR*-Dw	CATACGGGTATT**GAGCT**CATAATTCATT
*pclR∆105*	AGCCACTTATATT**GAG**CT**C**ATTTTTCCG
*pclR∆-10*	TTCATTATATC**GAGC**T**C**TGTCTAAGTAG
Up*pclA*	CTAATTTTTCGGC**G**AG**C**TCAT GTAATT
F*pclA∆173*	GAAAATTGTCGTTAG**AGC**TCGGAAAAATT
F*pclA∆203*	GAATAATATAAG**GA**GCT**C**TTAAGTGCAAA
F*pclA∆224*	GTGCAAAAACAGA**GCTC**ATTAATTGTT
R*pclA∆-10*	CAATGTACAACTATTTGAG**CTC**TTAATCAAT
*pclR*-*Nde*	ATGGAGGAAATACCC**A**TATGAGAAACCTTT
*pclR*-*Xho*-His	GACTTTTTTGAT**CT**C**GAG**TAAAGTATTGGA
Dw*1404-2*	CGGCTAGTTCATGTAATTTCATCCA
Int-*gfp*	CATCACCATCTAATTCAACAAG
F*era*-q	GATTGCCATCATGAGTGACAAGG
R*era*-q	AGTGTCCACTTCGCGAAGGGT
F*pclR*-q	CCAACCTCTATCGACTGGGCA
R*pclR*-q	CAGGAAGGTCAGGAAAAGGC
F*pclA*-q	GACGTGATGGTTCAGCTCCA
R*pclA*-q	GGATTTGTCACCGTAATTGT
*1622*A	AGTTCCTGATTGTATTCCCT
*1622*J	GAATAAGGATAATCTGATTTGGCA
F*1623*-q	GGGGGACAGTGGTTCTATCA
*1623*B	CGTAAATTTACATGAACAGTTGGG
− 40 M13	GTTTTCCCAGTCACGAC
Up*1404*	CTCCTAGATAGTATTTTATAGT
Dw*1404*	GAATTAGGGTTTCCATTAAGCGT
Up*1404*-2	CAATGTACAACTATTTGAGGCA
*1622*H	CGGATTAAACCTCTTGCAATTATACC
*1622*I	CAAATTCTTTAATTGTTGCTATTA

^a^Restriction sites are underlined, and the base changes that generate restriction sites are in bold.

### PclR activates the promoter of the *pclA* gene in bacterial cultures

By qRT-PCR assays and using total RNA from strains R6/pDLF*pclR* (high levels of *pclR* expression) and R6/pDLF*pclR-i* (low levels of *pclR* expression), we analysed the effect of PclR on the transcription of the *pclA* gene. Transcription of *pclA* was found to be higher (~ 3.4-fold) in the strain with high levels of *pclR* expression (Supplementary Table S5). Moreover, using total RNA from strains R6∆*mga*/pDLF*pclR* (high levels of *pclR* expression) and R6∆*mga*/pDLF*pclR-i* (low levels of *pclR* expression), we confirmed that the amount of *pclA* transcripts was higher (~ 4.5-fold) in the strain with high levels of *pclR* expression (Supplementary Table S6). These results indicated that PclR has a positive effect on the transcription of the *pclA* gene, both in the presence and in the absence of the Mga*Spn* regulator.

**Figure 3:**
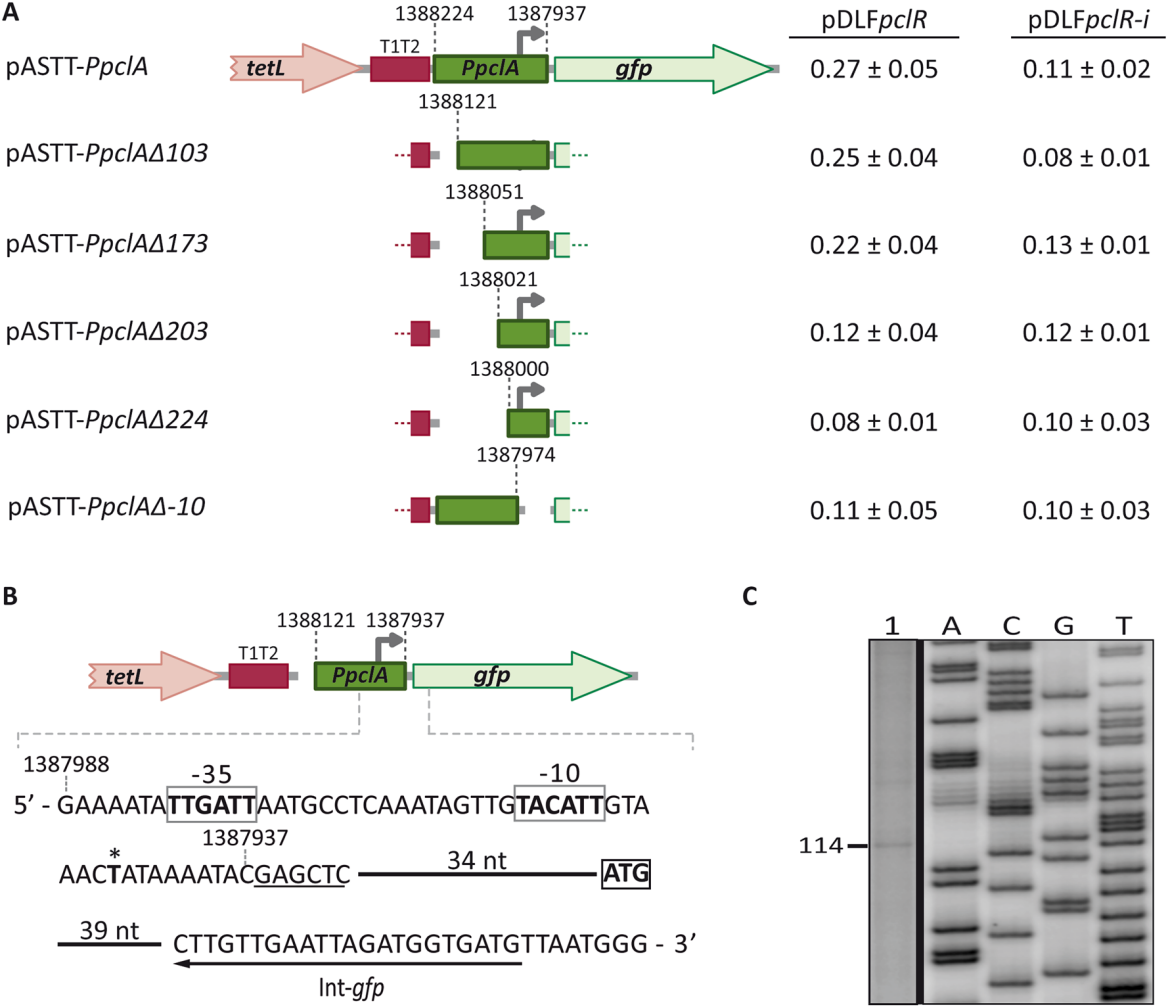
Identification of the *PpclA* promoter. **(A)** Fluorescence assays. Six regions of the R6 chromosome were amplified by PCR and inserted into the *Sac*I site of the promoter-probe vector pASTT. The coordinates of the inserted regions are indicated. The grey arrow represents the − 10 element of the *PpclA* promoter. See also legend to Fig. 2A. **(B)** Plasmid pASTT-*PpclA∆103*. The main sequence elements (− 35 box and − 10 box) of the *PpclA* promoter and the ATG translation start codon of the *gfp* gene are indicated. The *Sac*I site is underlined. The position of the Int-*gfp* oligonucleotide used for primer extension is shown. The asterisk indicates the transcription start site identified in this work. **(C)** Primer extension reaction using total RNA from strain R6∆*mga*/pDLF*pclR*/pASTT-*PpclA*∆103. The Int-*gfp* oligonucleotide was used as a primer. Dideoxy-mediated chain termination sequencing reactions (M13mp18 DNA and primer − 40 M13^36^) were run in the same gel as DNA size markers. The corresponding lanes (A, C, G, and T) came from the same gel but were taken at a lower exposure (delineation with dividing lines). See the full-length gel (high and low exposures) at the end of the Supplementary Information file. The size (in nucleotides) of the cDNA product is indicated on the left of the gel.

The ATG codon at coordinate 1,387,920 is likely the translation start site of the *pclA* gene (Fig. 1). Sequence analysis of the region located between coordinates 1,388,224 and 1,387,910 revealed the existence of a putative promoter (named *PpclA* herein), in which the − 35 (**TTGA**TT) and − 10 (**TA**C**A**T**T**) elements are separated by 17 nucleotides (optimal length). To analyse whether such a sequence had promoter activity, we constructed several transcriptional fusions based on the *gfp* reporter gene (Fig. 3A). First, we inserted a 288-bp DNA fragment (coordinates 1,388,224 to 1,387,937) into the promoter-probe vector pASTT and introduced the recombinant plasmid (pASTT-*PpclA*) into the pneumococcal R6 strain. Measuring the fluorescence of the cultures, we did not detect significant differences in *gfp* expression between R6/pASTT (0.07 ± 0.01 units; background level) and R6/pASTT-*PpclA* (0.08 ± 0.01 units). However, when pASTT-*PpclA* was introduced into R6∆*mga*/pDLF*pclR* (high levels of *pclR* expression) and R6∆*mga*/pDLF*pclR-i* (low levels of *pclR* expression), we detected a higher level of *gfp* expression (~ 2.5-fold) in the strain with high levels of *pclR* expression (Fig. 3A). Similar results were obtained with plasmids pASTT-*PpclA∆103* and pASTT-*PpclA∆173*, which allowed us to conclude that the 115-bp region between coordinates 1,388,051 and 1,387,937 contains a PclR-dependent promoter. No PclR-dependent promoter activity was detected (i) when the − 10 element of the *PpclA* promoter was deleted (from coordinate 1,387,974 to 1,387,937; plasmid pASTT-*PpclA∆-10*), and (ii) when a 30-bp region located upstream of the *PpclA* promoter was removed (from coordinate 1,388,051 to 1,388,021; plasmids pASTT-*Ppcl*A*∆203* and pASTT-*PpclA∆224*) (Figs. 1, 3A). Finally, by primer extension assays (Fig. 3C), we confirmed that the *PpclA* promoter located on pASTT-*PpclA∆103* (Fig. 3B) is functional. We used total RNA from strain R6∆*mga*/pDLF*pclR*/pASTT-*PpclA∆103* (high levels of *pclR* expression) and the oligonucleotide Int-*gfp*, which anneals to *gfp* transcripts (Fig. 3B). A cDNA product of 114 nucleotides was detected (Fig. 3C), which could correspond to a transcription initiation event at coordinate 1,387,946 (Figs. 1, 3B). This coordinate is located 7 nucleotides downstream of the − 10 element of the *PpclA* promoter. Taking together, we conclude that PclR activates transcription from the *PpclA* promoter. This activation requires sequences located between positions − 75 and − 105 of the *PpclA* promoter.

### PclR binds upstream of the *PpclA* core promoter

**Figure 4:**
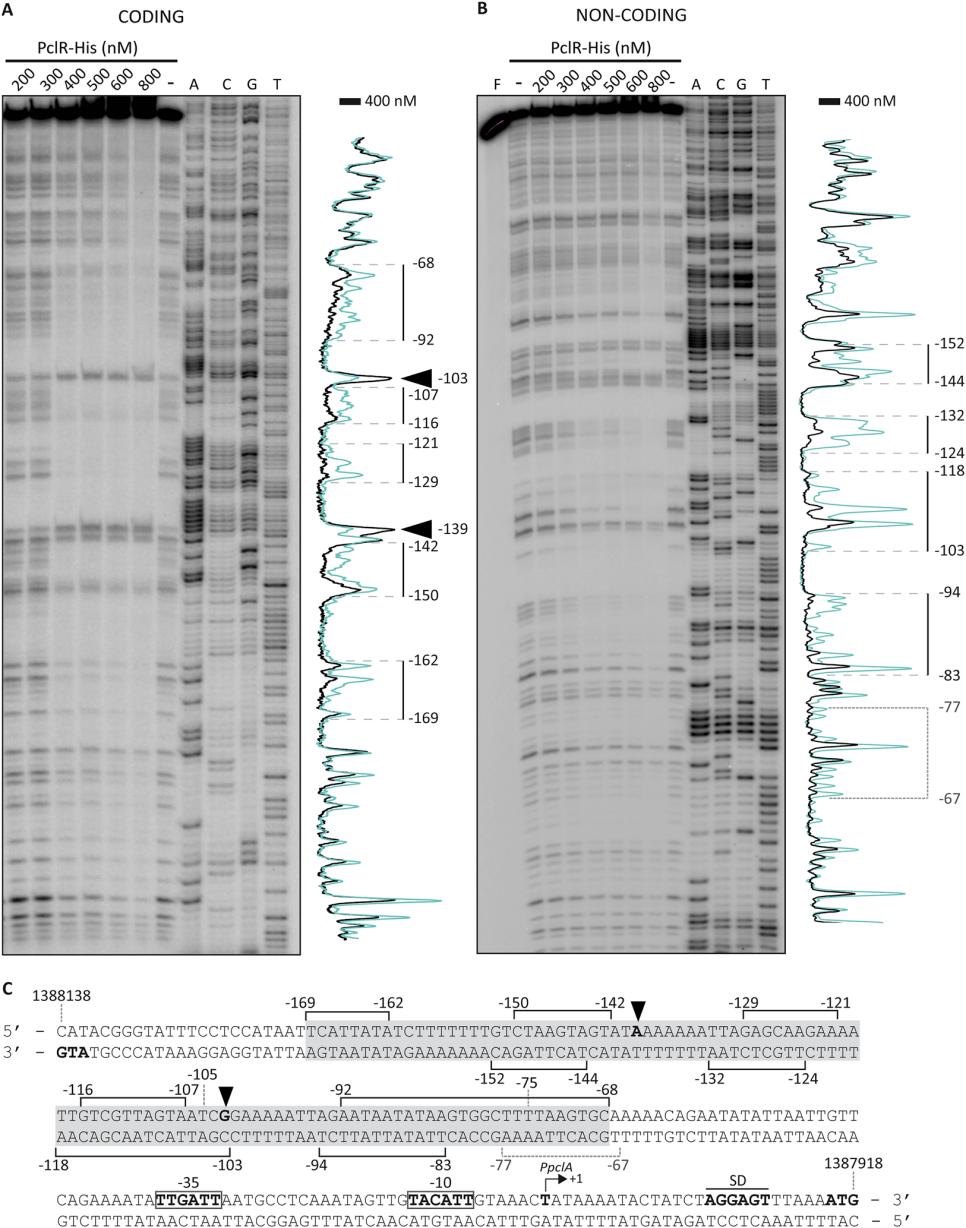
DNase I footprints of PclR-His-DNA complexes. **(A)** The 270-bp DNA fragment (coordinates 1,388,196 to 1,387,927) was ^32^P-labelled at the 5′ end of the coding strand (*pclA* gene) using the ^32^P-labelled Dw*1404* oligonucleotide. **(B)** The 281-bp DNA fragment (coordinates 1,388,232 to 1,387,952) was ^32^P-labelled at the 5′ end of the non-coding strand (relative to *pclA*) using the ^32^P-labelled Up*1404*-2 oligonucleotide. The labelled DNA (2 nM) was incubated with the indicated concentrations of PclR-His and then digested with DNase I. Non-digested DNA (F) and dideoxy-mediated chain termination sequencing reactions were run in the same gel (lanes A, C, G, T). In panel A, the sequence corresponds to the coding strand of the 270-bp DNA fragment (^32^P-labelled Dw*1404* oligonucleotide). In panel B, the sequence corresponds to the non-coding strand of the 281-bp DNA fragment (^32^P-labelled Up*1404*-2 oligonucleotide). Densitometer scans corresponding to DNA without PclR-His (blue line) and DNA with PclR-His (400 nM, black line) are shown. The protected regions are indicated with brackets. Arrowheads indicate positions that are slightly more sensitive to DNase I cleavage. The indicated positions are relative to the transcription start site of the *pclA* gene. **(C)** Nucleotide sequence of the region that spans coordinates 1,388,138 to 1,387,918 of the R6 chromosome. The − 35 and − 10 elements of the *PpclA* promoter are indicated. The transcription start site (+1 position), the putative Shine-Dalgarno sequence (SD), and the translation start codon (ATG) of the *pclA* gene are indicated. Brackets indicate regions protected against DNase I digestion. Black arrowheads indicate positions that are slightly more sensitive to DNase I cleavage. The grey box includes the site recognized by PclR-His.

By DNase I footprinting experiments, we analysed whether PclR recognized the *PpclA* promoter region. A 270-bp DNA fragment (coordinates 1,388,196 to 1,387,927) was radioactively labelled at the 5′-end of the coding strand. The labelled DNA (2 nM) was then incubated with increasing concentrations of a His-tagged version of the PclR protein (PclR-His) (Fig. 4A). At 400 nM of PclR-His, protections against DNase I digestion were observed at a particular region, from position − 169 to − 68 relative to the transcription initiation site of the *PpclA* promoter. Moreover, two positions located at − 139 and − 103 were more sensitive to DNase I cleavage (Fig. 4A). To determine the region protected by PclR-His on the non-coding strand, a 281-bp DNA fragment (coordinates 1,388,232 to 1,387,952) was radioactively labelled at the 5′-end of the non-coding strand (Fig. 4B). At 400 nM of PclR-His, major changes in DNase I sensitivity (diminished cleavages) were observed from position − 152 to − 83. These results indicated that PclR-His recognizes a site located between positions − 169 and − 68 of the *PpclA * promoter (Fig. 4C). This region contains the sequence (from position − 105 to − 75) that PclR needs to activate the *PpclA* promoter (Fig. 3A). Thus, we conclude that PclR activates transcription of the *pclA* gene by binding to a specific site upstream of the *PpclA* core promoter. Using the bend.it server (pongor.itk.ppke.hu/dna/bend_it.html), we calculated the bendability/curvature propensity plot of the 270-bp DNA fragment. The profile contains two potential intrinsic curvatures (~ 10–11 degrees per helical turn) within the PclR binding site (Supplementary Fig. S5). Intrinsic curvatures flanked by regions of bendability have been also predicted in DNA sites recognized by the Mga*Spn* transcriptional activator^17^.

On both DNA strands and at 800 nM of PclR-His (Fig. 4A,B), regions protected against DNase I digestion were observed along the DNA fragment, which suggested that, upon binding to the primary site, additional PclR-His units interacted with the adjacent DNA regions. This result is consistent with the ability of PclR-His to generate multimeric complexes on linear double-stranded DNAs (Supplementary Fig. S6A), a feature previously reported for the Mga*Spn* transcriptional regulator^17,24^. Specifically, we performed electrophoretic mobility shift assays (EMSAs) with the 270-pb DNA fragment that had been used in the DNase I footprinting assay. As shown in Supplementary Fig. S6A, the ^32^P-labelled DNA was incubated with different concentrations of PclR-His in the presence of non-labelled competitor calf thymus DNA. Free and bound DNAs were separated by electrophoresis on a native polyacrylamide (6%) gel. At 200 nM of PclR-His, free DNA and four protein-DNA complexes were detected. In addition, as the protein concentration was increased, such complexes disappeared and higher-order complexes appeared. This pattern of complexes suggested that multiple protein units bind orderly on the same linear DNA molecule.

### PclR and Mga*Spn* have different DNA-binding specificities

**Figure 5:**
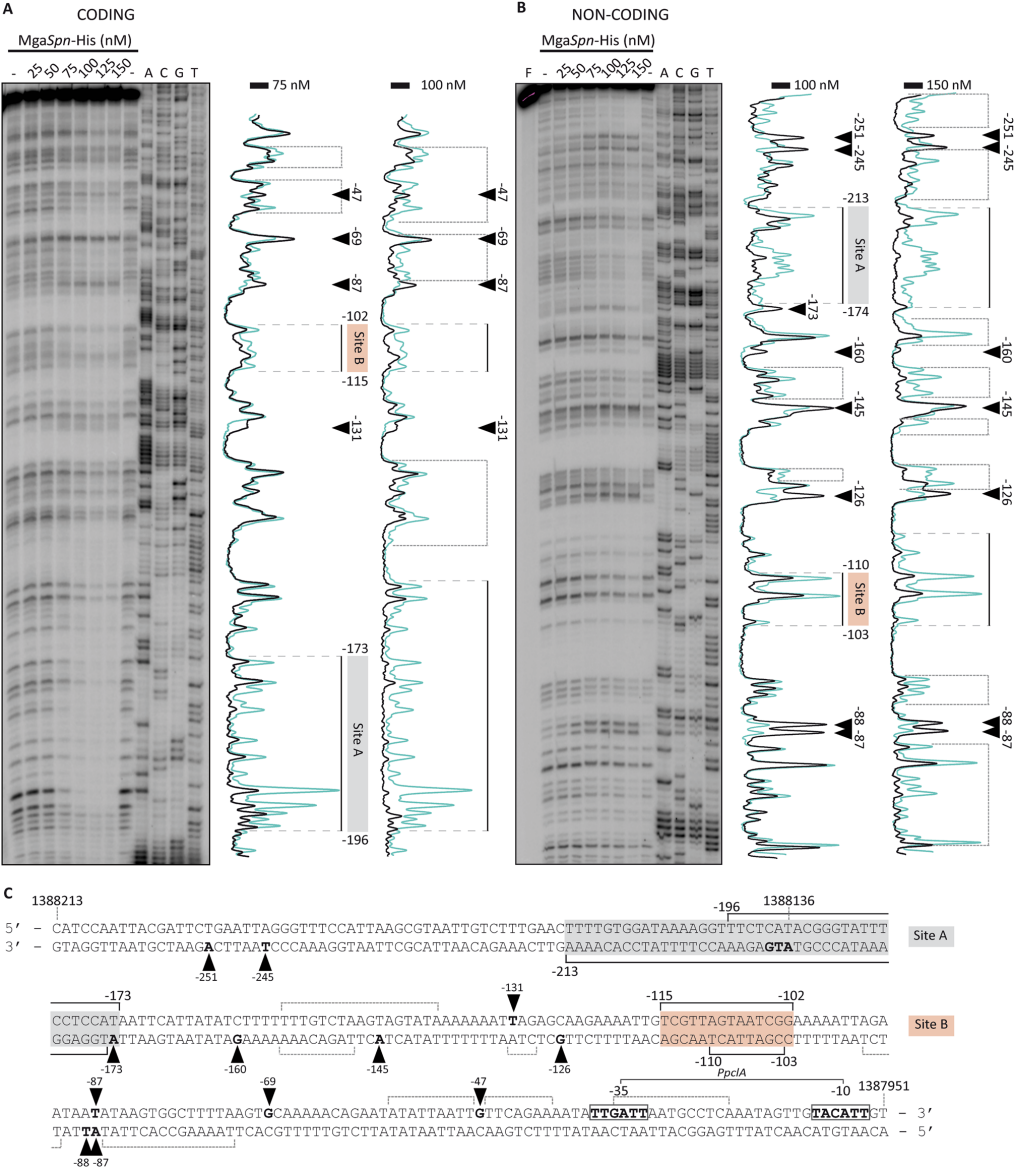
DNase I footprints of Mga*Spn*-His-DNA complexes. **(A)** Binding of Mga*Spn*-His to the 270-bp DNA fragment (coordinates 1,388,196 to 1,387,927), which was ^32^P-labelled at the 5′ end of the coding strand (relative to *pclA*) using the ^32^P-labelled Dw*1404* oligonucleotide. **(B)** Binding of Mga*Spn*-His to the 281-bp DNA fragment (coordinates 1,388,232 to 1,387,952), which was ^32^P-labelled at the 5′ end of the non-coding strand (relative to *pclA*) using the ^32^P-labelled Up*1404*-2 oligonucleotide. Non-digested DNA (F) and dideoxy-mediated chain termination sequencing reactions were run in the same gel (lanes A, C, G, T). In panel A, the sequence corresponds to the coding strand of the 270-bp DNA fragment (^32^P-labelled Dw*1404* oligonucleotide). In panel B, the sequence corresponds to the non-coding strand of the 281-bp DNA fragment (^32^P-labelled Up*1404*-2 oligonucleotide). Densitometer scans corresponding to DNA without Mga*Spn*-His (blue line) and DNA with Mga*Spn*-His (black line) are shown. Brackets represent the Mga*Spn*-His protected regions. Positions more sensitive to DNase I cleavage are indicated with arrowheads. The indicated positions are relative to the transcription start site (+1 position) of the *pclA* gene. **(C)** Nucleotide sequence of the region that spans coordinates 1,388,213 to 1,387,951 of the R6 chromosome. The − 35 and − 10 elements of the *PpclA* promoter are indicated. Mga*Spn*-His protected regions (brackets) and positions more sensitive to DNase I cleavage (arrowheads) are indicated. The two sites recognized preferentially by Mga*Spn*-His (Sites A and B) are shown.

**Figure 6:**
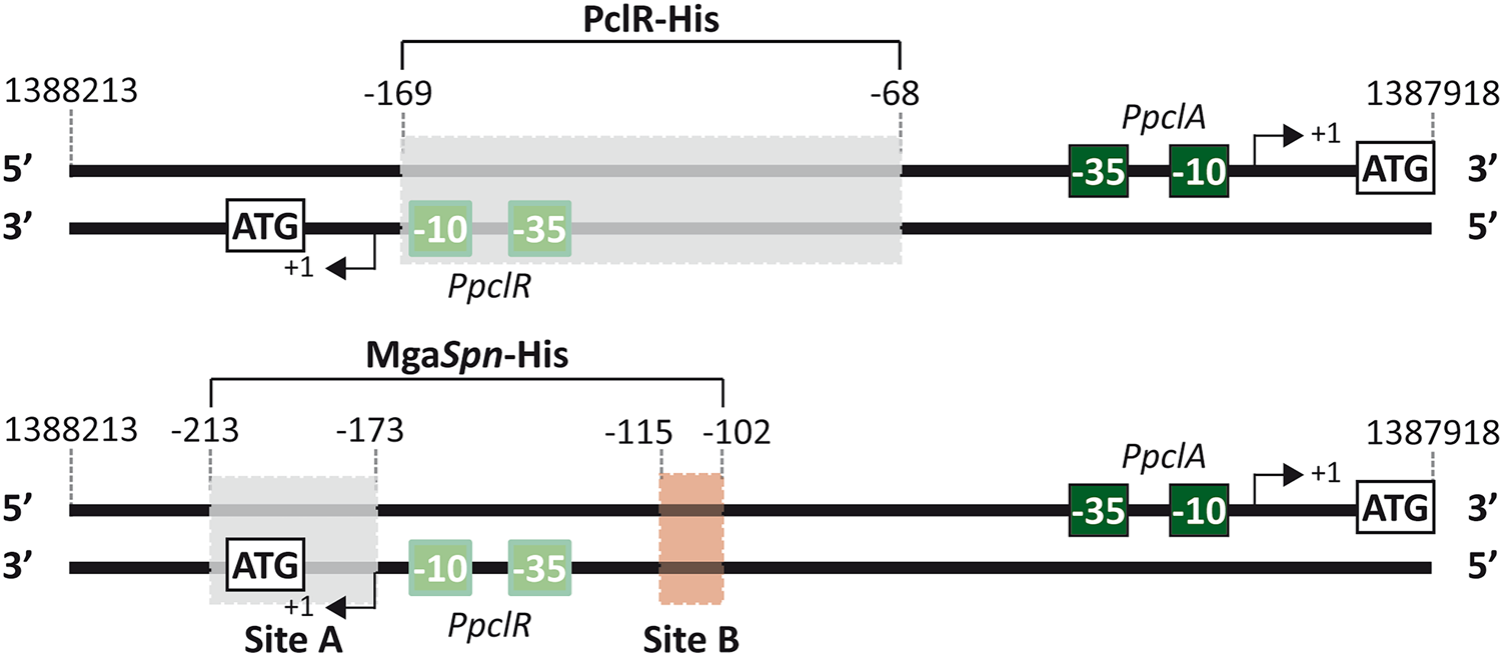
Sites recognized by PclR-His and Mga*Spn*-His in the region that contains the *PpclA* and *PpclR* promoters. The sites were defined by DNase I footprinting assays (see Figs. 4 and 5). The location of the main elements of each promoter (− 35 and − 10 boxes), as well as the transcription start site (position +1) and the translation initiation codon (ATG) of the *pclA* and *pclR* genes are indicated. The − 68, − 102, − 115, − 169, − 173 and − 213 positions are relative to the transcription start site of the *pclA* gene.

According to EMBOSS Needle Pairwise Sequence Alignment^25,26^, the N-terminal regions (first 170 amino acids) of PclR and Mga*Spn* share high sequence similarity (66.5% of similarity and 50% of identity). Both regions contain two predicted helix-turn-helix DNA-binding domains, the so-called HTH_Mga (residues 6 to 65) and Mga (residues 72 to 158) domains (Supplementary Figs. S1, S2, and S3). To know whether Mga*Spn* recognized the *PpclA* promoter region, we performed DNase I footprinting assays using Mga*Spn*-His and the 270-bp DNA fragment. The 270-bp DNA fragment was radioactively labelled at the 5′-end of the coding strand (Fig. 5A). At 75 nM of Mga*Spn*-His, diminished DNase I cleavages were observed from position − 173 to − 196, and from − 102 to − 115. Moreover, positions − 47, − 69, − 87, and − 131 were slightly more sensitive to DNase I digestion (Fig. 5A,C). This result was confirmed in shorter electrophoretic runs (Supplementary Fig. S7). At higher Mga*Spn*-His concentrations, protections against DNase I digestion were observed along the entire DNA fragment (Fig. 5A), which is consistent with the pattern of protein-DNA complexes observed by EMSA (Supplementary Fig. S6B), and with the ability of Mga*Spn* to form multimeric complexes on linear DNA^17^. The region protected by Mga*Spn*-His on the non-coding strand was defined using the 281-bp DNA fragment (Fig. 5B). At 100 nM of Mga*Spn*-His, diminished cleavages were mostly observed from position − 174 to − 213, and from − 103 to − 110. In addition, positions − 87, − 88, − 126, − 145, − 160, − 173, − 245 and − 251 were more sensitive to DNase I digestion (Fig. 5B,C). These results showed that PclR-His and Mga*Spn*-His recognize different sites on the *PpclA* promoter region (Fig. 6). Mga*Spn*-His binds preferentially to two sites: Site A (from − 173 to − 213) and Site B (from − 102 to − 115). Site A is adjacent to the region recognized by PclR-His (from − 68 to − 169) and Site B is included within such a region. Next, we analysed whether Mga*Spn* influenced the expression of the *pclA* gene. Specifically, by qRT-PCR assays, we determined the relative expression of the *pclA* gene in two strains: R6∆*mga*/pDL*PsulA*::*mga* (plasmid-encoded Mga*Spn*) and R6∆*mga*/pDL287 (absence of Mga*Spn*). As shown in Supplementary Table S7, plasmid-encoded Mga*Spn* had no significant effect on the intracellular levels of *pclA* transcripts. Plasmid pDL*PsulA*::*mga* had been used previously to demonstrate that plasmid-encoded Mga*Spn* activates the *P1623B* promoter^15^. Thus, the function (if any) of the interaction of Mga*Spn*-His with the sites A and B (Fig. 6) remains unknown.

Mga*Spn* activates the transcription of the *spr1623-spr1626* operon by binding to a specific site upstream of the *P1623B* promoter (positions − 60 to − 99)^15,17^. By DNase I footprinting assays, we also analysed whether PclR-His recognized the *P1623B* promoter region. We used a 222-bp DNA fragment (coordinates 1,598,298 to 1,598,519) that contains the *P1623B* promoter and the site recognized by Mga*Spn*^17^. Specific regions protected against DNase I digestion were not detected (Supplementary Fig. S8), indicating that PclR-His does not recognize a specific site on the *P1623B* promoter region. This result correlated with the inability of PclR to influence the activity of the *P1623B* promoter. By qRT-PCR assays, we found similar levels of *spr1623* transcripts in strains that produce different levels of PclR: R6/pDLF*pclR* (high levels of *pclR* expression) versus R6/pDLF*pclR-i* (low levels of *pclR* expression) (Supplementary Table S8), and R6∆*mga*/pDLF*pclR* (high levels of *pclR* expression) versus R6∆*mga*/pDLF*pclR-i* (low levels of *pclR* expression) (Supplementary Table S9).

Taken together, we conclude that PclR and Mga*Spn* have different DNA-binding specificities. They recognize different sites on the *PpclA* promoter region. Moreover, unlike Mga*Spn*, PclR does not bind to the *P1623B* promoter region. In agreement with these results, Mga*Spn* does not affect the activity of the *PpclA* promoter, and PclR does not affect the activity of the *P1623B* promoter.

## Discussion

*S. pneumoniae* is an opportunistic pathogen able to proliferate in different niches of the human host. Its adaptation to new environments and host-imposed stresses partially relies on the activity of specific transcriptional regulators. The genome of the pneumococcal R6 strain has several gene clusters that are absent from other strains. One of these clusters contains two divergent genes, *pclA*, which encodes a putative cell surface protein^12^, and *pclR*, whose function has been investigated in this work. We have identified the promoter of each gene (*PpclA* and *PpclR*) and demonstrated that PclR functions as a transcriptional activator. It stimulates *pclA* transcription by binding to a specific site upstream of the *PpclA* core promoter. PclA is a collagen-like protein, which contains the peptidoglycan anchor LPXTG motif and several GXY amino acid repeats^12^. This repeating pattern is the most typical feature in the molecular architecture of bacterial collagen-like proteins^37^. In pathogenic streptococci, surface-exposed collagen-like proteins have been associated with processes of colonization, biofilm formation, and evasion of the host immune response^38^. In the case of PclA, Paterson *et al.*^12^ reported that a *pclA* deletion mutant strain is defective in adherence and invasion of nasopharyngeal and epithelial cells in vitro. Thus, we speculate that PclR could have a regulatory role during pneumococcal colonization. Using the EMBOSS Needle Pairwise Sequence Alignment program^25,26^, we have found that PclR has sequence similarity (40.4%) to the Mga global regulator (530 residues; GenBank AAT87855.1) of the Gram-positive bacterium *S. pyogenes* (Group A *Streptococcus*; GAS). It has been reported that Mga regulates positively the transcription of the *scl1* gene (also known as *sclA*)^39–41^. This gene encodes a collagen-like surface protein (Scl1) that interacts with integrins, cellular fibronectin, and laminin^42–44^. Moreover, it has been shown that Scl1 mediates GAS adherence to and internalization by human pharyngeal epithelial cells, playing an important role in pathogenesis^43^.

DNA rearrangements and gene acquisition are natural strategies for the generation of genetic diversity in *S. pneumoniae*, a feature that has been recently shown to be increased by the presence of temperate bacteriophages integrated into different regions of the pneumococcal chromosome^45^. It has been reported that the gene content between pairs of pneumococcal isolates can diverge by as much as 30%^46^. The sequences of the pneumococcal TIGR4 and R6 genomes were published in 2001^9,10^. A comparison of the two sequences revealed the existence of strain-specific genes, many of which are organized in clusters. Specifically, the TIGR4 genome has twelve gene clusters (~ 7% of the total genome) that are not present in R6, and the R6 genome has six gene clusters (~ 3% of the total genome) that are absent from TIGR4^11^. PCR analyses of the distribution of the R6-specific *pclA*-*pclR* gene cluster in a collection of clinical isolates revealed that many of such isolates lacked both genes (~ 60% of the strains examined)^12^. Subsequently, *pclA* was found to be associated with Pneumococcal Molecular Epidemiology Network (PMEN) clones^13^. Clones included in the PMEN are resistant to one or more antibiotics that are in wide clinical use. Moreover, they have a wide geographic distribution (https://www.pneumogen.net/pmen). Now, we have analysed whether the *pclR* gene was present in the 24 pneumococcal genomes shown in Supplementary Table S10. Such genomes are fully sequenced and assembled (NCBI database). Moreover, they encode a highly conserved Mga*Spn* regulator^15,21^. Using the BLASTP protein sequence alignment program^47^, we have found that only nine out of the 24 genomes encode PclR: strains ATCC 700669, A026, D39, JJA, INV104, ST556, Taiwan19F14, TCH8431/19A and 70585. The PclR regulator of these strains is identical or almost identical to the PclR regulator encoded by the R6 genome (Supplementary Table S10). Like R6, the nine genomes also encode PclA.

A study based on RNA-seq revealed profound changes in the relative amount of the RNAs synthesized by the pneumococcal D39V strain under a wide range of infection-relevant conditions. The expression data as well as the co-expression matrix were published in the PneumoExpress database (https://veeninglab.com/pneumoexpress)^48^. The D39V genome contains the *pclA-pclR* gene cluster (genes *SPV_1376* and *SPV_1377* in D39V). Searching in PneumoExpress, we have found that the highest expression level of *pclA* and *pclR* corresponds to bacteria grown in nose mimicking conditions, which simulate colonization. Both genes were also highly expressed in bacteria grown in lung mimicking conditions, which simulate pneumonia, and in cerebrospinal fluid-mimicking conditions from 37 to 40 °C, which simulate meningeal fever. In the case of the *mgaSpn* regulatory gene (*SPV_1587*) and its target operon *spr1623-spr1626* (*SPV_1588-SPV_1591*), the highest expression level corresponds also to bacteria grown in nose mimicking conditions. Hence, the expression data suggest that PclR and Mga*Spn* could play a significant role during nasopharyngeal colonization. Previous studies performed by Hemsley *et al.*^16^ showed that a *mgaSpn* deletion mutant strain was attenuated for both nasopharyngeal carriage and pneumonia in murine infection models. Concerning the expression of the *mgaSpn* and *pclR* regulatory genes under standard laboratory conditions (this work), transcription of both genes was found to be higher in the logarithmic phase compared to the stationary phase. Most of the transcription processes in exponentially growing pneumococcal bacteria are initiated by the RNA polymerase that contains the housekeeping sigma factor SigA, also known as RpoD and σ43. In the promoters recognized by the housekeeping factor, the consensus sequence of the − 10 element is 5′-TATAAT-3′, which is present in the promoter of *mgaSpn* (*Pmga*) and the promoter of *pclR* (*PpclR*). It has been shown that SigA recognizes the *Pmga* promoter in vitro^49^.

The pneumococcal Mga*Spn* transcriptional regulator is a member of the Mga/AtxA family^17–19^, which also includes the global regulator MafR of *E. faecalis*^20^. Here we have shown that PclR shares some features with Mga*Spn*. These proteins have the same size (494–493 residues), exhibit a high degree of sequence similarity (60%), and have the same organization of predicted functional domains, including two N-terminal helix-turn-helix DNA-binding motifs. Furthermore, PclR can generate multimeric complexes on linear double-stranded DNA fragments, a feature reported first for Mga*Spn*^17^ and later on for MafR^50^. Regarding their mechanism to activate transcription from specific promoters, both proteins stimulate transcription by binding to a specific site upstream of the core promoter. PclR recognizes a site upstream of the *PpclA* promoter (positions − 68 to − 169), and Mga*Spn* activates transcription of a four-gene operon (*spr1623*-*spr1626*) by binding to a site upstream of the *P1623B* promoter (positions − 60 to − 99)^17^. Nevertheless, despite these similarities, we have shown that PclR and Mga*Spn* have different DNA-binding specificities. PclR does not bind to the site recognized by Mga*Spn* on the *P1623B* promoter region, and Mga*Spn* does not bind to the site recognized by PclR on the *PpclA* promoter region. As a consequence, PclR does not influence the expression of the *spr1623* gene, and Mga*Spn* does not influence the expression of the *pclA* gene.

In summary, the *pclA-pclR* gene cluster of the pneumococcal R6 strain is not present in all strains of the species. Our present work demonstrates that PclR is a transcriptional activator of the *pclA* gene (collagen-like protein). PclR recognizes a specific DNA site upstream of the *PpclA* core promoter. Moreover, PclR is homologous to the Mga*Spn* transcriptional regulator, which is also encoded by the R6 genome. Our study shows that PclR and Mga*Spn* have similar DNA-binding properties but different DNA-binding specificities.

## Materials and methods

### Oligonucleotides, bacterial strains, and plasmids

The oligonucleotides used in this work are listed in Table 1. *S. pneumoniae* strains R6^10^ and R6∆*mga*^15^ were used. R6∆*mga* lacks the *mgaSpn* regulatory gene. The pneumococcal strains R6∆*mga*/pDL287 (absence of Mga*Spn*) and R6∆*mga*/pDL*PsulA*::*mga* (plasmid-encoded Mga*Spn*) were described previously^15^. Plasmid pDLF is a constitutive expression vector that carries a kanamycin resistance gene^20^. This vector has an engineered unique restriction site for *Sph*I downstream of the enterococcal *P2493* promoter^34^. Plasmids pDLF*pclR* and pDLF*pclR-i* are pDLF derivatives. For their construction, a 1594-bp region of the R6 chromosome was amplified by PCR using the *FpclR* and *RpclR* oligonucleotides. After *Sph*I digestion, the 1561-bp restriction fragment was inserted into the *Sph*I site of pDLF in both orientations, being pDLF*pclR* the recombinant plasmid that carries the gene *pclR* under the control of the *P2493* promoter. Plasmid pASTT is a promoter-probe vector based on the *gfp* reporter gene^51^. It is a pAST derivative^34^ and carries a tetracycline resistance gene. The following pASTT-derivatives were constructed in this work. In all cases, a region of the R6 chromosome was amplified by PCR using the indicated primers. Then, the PCR product was digested with *Sac*I, and the restriction fragment was ligated to the *Sac*I-linearized pASTT vector: (a) pASTT-*PpclR* (primers Dw*pclA* and *pclR*-Dw, 190-bp restriction fragment), (b) pASTT-*PpclR∆105* (primers *pclR∆105* and *pclR*-Dw, 85-bp restriction fragment), (c) pASTT-*PpclR∆-10* (primers Dw*pclA* and *pclR∆-10*, 169-bp restriction fragment), (d) pASTT-*PpclA* (primers Up*pclA* and Dw*pclA*, 292-bp restriction fragment), (e) pASTT-*PpclA∆103* (primers *pclR*-Dw and Dw*pclA*, 190-bp restriction fragment), (f) pASTT-*PpclA∆173* (primers F*pclA∆173* and Dw*pclA*, 119-bp restriction fragment), (g) pASTT-*PpclA∆203* (primers F*pclA∆203* and Dw*pclA*, 91-bp restriction fragment), (h) pASTT-*PpclA∆224* (F*pclA∆224* and Dw*pclA*, 70-bp restriction fragment) and (i) pASTT-*PpclA∆-10* (primers Up*pclA* and R*pclA∆-10*, 255-bp restriction fragment). For protein overproduction, an inducible expression system based on the *Escherichia coli* strain BL21(DE3) (a gift of F. W. Studier) and the plasmid vector pET24b (Novagen) was used. This strain carries the gene for T7 RNA polymerase under the control of the *lacUV5* promoter^52^, which is inducible by isopropyl β-D-1-thiogalactopyranoside (IPTG). Vector pET24b is based on the phi10 promoter recognized by the T7 RNA polymerase. Plasmid pET24b-*pclR*-His encodes the PclR-His protein, which carries the Leu-Glu-6xHis peptide fused to its C-terminus. For its construction, a 1517-bp region of the R6 chromosome was amplified by PCR using the *pclR*-*Nde* and *pclR*-*Xho*-His oligonucleotides. The amplified DNA was digested with *Nde*I and *Xho*I, and the 1484-bp digestion product was inserted into pET24b. Plasmid pET24b-*mgaSpn*-His encodes the Mga*Spn*-His protein^15^.

### Growth and transformation of bacteria

Pneumococcal cells were grown in AGCH medium^34,53^ supplemented with 0.3% sucrose and 0.2% yeast extract, at 37 °C in a static water bath. For plasmid-harbouring cells, the medium was supplemented with kanamycin (50 µg/ml; pDLF derivatives) and/or tetracycline (1 µg/ml; pASTT derivatives). The protocol used for natural transformation of *S. pneumoniae* was described previously^33^. *E. coli* cells carrying a pET24b derivative were grown in tryptone-yeast extract (TY) medium supplemented with kanamycin (30 µg/ml), at 37 °C in a shaking water bath. The protocol used to transform *E. coli* by electroporation was described previously^54^.

### Overproduction and purification of His-tagged proteins

*E. coli* strains BL21(DE3)/pET24b-*mgaSpn*-His^15^ and BL21(DE3)/pET24b-*pclR*-His (this work, see above) were used. The protocols used to overproduce and purify the Mga*Spn*-His protein were described previously^15^. Mga*Spn*-His purification involved the use of a HisTrap HP column (GE Healthcare) and a HiLoad Superdex 200 gel filtration column (Amersham). For overproduction and purification of the PclR-His protein, the protocols reported for MafR-His^50^ were used. Basically, PclR-His purification included the following steps: (i) precipitation of nucleic acids with polyethyleneimine (PEI) (0.2%) in the presence of NaCl (300 mM). The ionic strength at which PEI precipitation was done was low enough to recover PclR-His in the PEI pellet, (ii) elution of PclR-His from the PEI pellet using a higher ionic strength buffer (700 mM NaCl), (iii) precipitation of the eluted proteins with 70% saturated ammonium sulphate, and (iv) fast-pressure liquid chromatography (Biologic Duoflow, Bio-Rad) on a nickel affinity column (HisTrap HP) (Supplementary Fig. S9). Protein concentration was determined using a NanoDrop ND-2000 Spectrophotometer (Thermo Scientific).

### DNA and RNA isolation

Genomic DNA from *S. pneumoniae* was prepared as reported^53^. Plasmid DNA was prepared using the High Pure Plasmid Isolation Kit (Roche Applied Science) as described^34^. Total RNA was isolated using the RNeasy Mini Kit (QIAGEN). Cultures were processed as specified by the supplier, except that cells were resuspended in a buffer that contained 50 mM Tris–HCl, pH 7.6, 1 mM EDTA, 50 mM NaCl, and 0.1% deoxycholate. Then, cells were incubated at 37 °C for 5 min (cell lysis). The integrity of rRNAs was analysed by agarose gel electrophoresis. RNA concentration was determined using a NanoDrop ND-2000 Spectrophotometer.

### Polymerase chain reaction (PCR) and quantitative RT-PCR (qRT-PCR)

Phusion High-Fidelity DNA Polymerase (Thermo Scientific) was used for all PCR applications as reported^34^. PCR products were purified with the QIAquick PCR Purification Kit (QIAGEN). In the qRT-PCR assays, for each strain, total RNA was isolated from three independent bacterial cultures. Then, from each RNA preparation, cDNA was synthesized. For cDNA synthesis with random primers, the iScript Select cDNA Synthesis Kit (Bio-Rad) was used as described previously^20^. To rule out the presence of genomic DNA in the RNA preparations, reactions without adding reverse transcriptase were performed. Quantitative PCRs were carried out using the iQ SYBR Green Supermix (Bio-Rad) and an iCycler Thermal Cycler (Bio-Rad) as reported^20^. From each cDNA sample, three PCRs per gene (gene of interest and internal control gene) were performed. Data were analysed with the iQ^TM^5 Optical System Software. Relative quantification of gene expression was performed using the comparative *C*_T_ method^32^. The *era* gene (*spr0871*) was used as the internal control gene (oligonucleotides F*era*-q and R*era*-q). The oligonucleotides used to determine the relative expression of the *pclR* (F*pclR*-q and R*pclR*-q)*, pclA* (F*pclA*-q and R*pclA*-q), *mgaSpn* (*1622*A and *1622* J) and *spr1623* (F*1623*-q and *1623*B) are shown in Table 1. The threshold cycle values (*C*_T_) of the gene of interest and the internal control gene were used to calculate 2^−Δ*C*T^, where Δ*C*_T_ = *C*_T_ gene of interest—*C*_T_ internal control. In general, for each cDNA sample (total three), the mean *C*_T_ from the three PCRs for the gene of interest, the mean *C*_T_ from the three PCRs for the internal control gene, and the 2^−Δ*C*T^ value were calculated. Then, the mean ± standard deviation of the three 2^−Δ*C*T^ values was calculated. The differences between two groups were analysed using a Student´s *t*-test (paired, two-tailed). For the gene of interest, the fold change in expression (FC) in one strain compared to another was obtained by dividing the corresponding mean 2^−Δ*C*T^ values. The results of these analyses are shown in Supplementary Tables S1 to S9 and Supplementary Fig. S10.

### Primer extension

The ThermoScript Reverse Transcriptase enzyme (Invitrogen) and [α-^32^P]-dATP (3000 Ci/mmol; PerkinElmer) were used as reported^15^. Basically, primer extension reactions (20 µl) contained 1 pmol of the indicated oligonucleotide and 2.5–15 µg of total RNA from the indicated strain. To anneal the primer with the transcript, samples were incubated at 65 °C for 5 min. Extension reactions were carried out at 55 °C for 45 min. After heating at 85 °C for 5 min, non-incorporated [α-^32^P]-dATP was removed using Illustra MicroSpin™ G-25 columns (GE Healthcare). Samples were ethanol precipitated as described^51^. cDNA products were analysed by sequencing gel (8 M urea, 6% polyacrylamide) electrophoresis. As DNA size markers, dideoxy-sequencing reactions were carried out using M13mp18 DNA, primer − 40 M13^36^, and the Sequenase Version 2.0 DNA Sequencing kit (USB Corporation). Labelled products were visualized using a Fujifilm Image Analyser FLA-3000.

### Fluorescence assays

Pneumococcal cells harbouring a pASTT derivative were grown as indicated above to an optical density at 650 nm (OD_650_) of 0.3–0.4 (exponential phase). Then, cultures were processed as reported^51^. Fluorescence intensity was measured using a Thermo Scientific Varioskan Flash instrument.

### DNase I footprinting assays

Oligonucleotides were ^32^P-labelled at the 5′-end as described^17^. PCR amplification using a ^32^P-labelled oligonucleotide was used to obtain double-stranded DNA fragments labelled at the 5′-end of one of the strands. Three regions of the R6 chromosome were amplified by PCR: (a) a 270-bp region (coordinates 1,388,196 to 1,387,927) using the Up*1404* and Dw*1404* oligonucleotides, (b) a 281-bp region (coordinates 1,388,232 to 1,387,952) using the Up*1404*-2 and Dw*1404*-2 oligonucleotides, and (c) a 222-bp region (coordinates 1,598,298 to 1,598,519) using the *1622*H and *1622*I oligonucleotides. Binding reactions and DNase I digestion were performed as described^51^. Samples were analysed by sequencing gel (6% polyacrylamide, 8 M urea) electrophoresis. Labelled products were visualized using a Fujifilm Image Analyser FLA-3000 and the intensity of the bands was quantified using the Quantity One software (Bio-Rad).

### Electrophoretic mobility shift assays

Binding reactions were performed as described^50^. When indicated, non-labelled competitor calf thymus DNA and ^32^P-labelled DNA were added simultaneously to the binding reaction. Reaction mixtures were analysed by electrophoresis on native polyacrylamide (6%) gels.

## Supplementary Information

Supplementary Information.

## Data Availability

All data generated and analysed during this study are included in this Manuscript and the Supplementary Information file. The sequences of genes and proteins analysed in the current study are available in the NCBI database: Locus_tag = SPR_RS06975 (old_locus_tag = spr1404). Gene ID: 60,234,404. https://www.ncbi.nlm.nih.gov/gene/?term=SPR_RS06975. https://www.ncbi.nlm.nih.gov/protein/WP_001245194.1. Locus_tag = SPR_RS08055 (old_locus_tag = spr1622). Gene ID: 60,233,139. https://www.ncbi.nlm.nih.gov/gene/?term=SPR_RS08055. https://www.ncbi.nlm.nih.gov/protein/WP_001205275.1.

## References

[1] Vernikos G, Medini D, Riley DR, Tettelin H (2015). Ten years of pan-genome analyses. Curr. Opin. Microbiol..

[2] Brooks LRK, Mias GI (2018). *Streptococcus pneumoniae*´s virulence and host immunity: Aging, diagnostics, and prevention. Front. Immunol..

[3] Loughran AJ, Orihuela CJ, Tuomanen EI (2019). *Streptococcus pneumoniae*: Invasion and Inflammation. Microbiol. Spectr..

[4] Bravo A, Ruiz-Cruz S, Alkorta I, Espinosa M (2018). When humans met superbugs: Strategies to tackle bacterial resistances to antibiotics. Biomol. Concepts.

[5] Hiller NL, Sá-Leao R (2018). Puzzling over the pneumococcal pangenome. Front. Microbiol..

[6] Hiller NL (2007). Comparative genomic analyses of seventeen *Streptococcus pneumoniae* strains: Insights into the pneumococcal supragenome. J. Bacteriol..

[7] Donati C (2010). Structure and dynamics of the pan-genome of *Streptococcus pneumoniae* and closely related species. Genome Biol..

[8] Chaguza C, Cornick JE, Everett DB (2015). Mechanisms and impact of genetic recombination in the evolution of *Streptococcus pneumoniae*. Comput. Struct. Biotechnol. J..

[9] Tettelin H (2001). Complete genome sequence of a virulent isolate of *Streptococcus pneumoniae*. Science.

[10] Hoskins J (2001). Genome of the bacterium *Streptococcus pneumoniae* strain R6. J. Bacteriol..

[11] Brückner R, Nuhn M, Reichmann P, Weber B, Hakenbeck R (2004). Mosaic genes and mosaic chromosomes-genomic variation in *Streptococcus pneumoniae*. Int. J. Med. Microbiol..

[12] Paterson GK, Nieminen L, Jefferies JM, Mitchell TJ (2008). PclA, a pneumococcal collagen-like protein with selected strain distribution, contributes to adherence and invasion of host cells. FEMS Microbiol. Lett..

[13] Imai S (2011). Distribution and clonal relationship of cell surface virulence genes among *Streptococcus pneumoniae* isolates in Japan. Clin. Microbiol. Infect..

[14] McGee L (2001). Nomenclature of major antimicrobial-resistant clones of *Streptococcus pneumoniae* defined by the pneumococcal molecular epidemiology network. J. Clin. Microbiol..

[15] Solano-Collado V, Espinosa M, Bravo A (2012). Activator role of the pneumococcal Mga-like virulence transcriptional regulator. J. Bacteriol..

[16] Hemsley C, Joyce E, Hava DL, Kawale A, Camilli A (2003). MgrA, an orthologue of Mga, acts as a transcriptional repressor of the genes within the *rlrA* pathogenicity islet in *Streptococcus pneumoniae*. J. Bacteriol..

[17] Solano-Collado V, Lurz R, Espinosa M, Bravo A (2013). The pneumococcal Mga*Spn* virulence transcriptional regulator generates multimeric complexes on linear double-stranded DNA. Nucleic Acids Res..

[18] Hondorp ER (2013). PTS phosphorylation of Mga modulates regulon expression and virulence in the group A *Streptococcus*. Mol. Microbiol..

[19] Hammerstrom TG (2015). Crystal structure of *Bacillus anthracis* virulence regulator AtxA and effects of phosphorylated histidines on multimerization and activity. Mol. Microbiol..

[20] Ruiz-Cruz S, Espinosa M, Goldmann O, Bravo A (2016). Global regulation of gene expression by the MafR protein of *Enterococcus faecalis*. Front. Microbiol..

[21] Gámez G (2018). The variome of pneumococcal virulence factors and regulators. BMC Genomics.

[22] Hava DL, Camilli A (2002). Large-scale identification of serotype 4 *Streptococcus pneumoniae* virulence factors. Mol. Microbiol..

[23] Paterson GK, Mitchell TJ (2006). The role of *Streptococcus pneumoniae* sortase A in colonisation and pathogenesis. Microbes Infect..

[24] Solano-Collado V, Hüttener M, Espinosa M, Juárez A, Bravo A (2016). Mga*Spn* and H-NS: Two unrelated global regulators with similar DNA-binding properties. Front. Mol. Biosci..

[25] Rice P, Longden I, Bleasby A (2000). EMBOSS: The European molecular biology open software suite. Trends Genet..

[26] Madeira F (2019). The EMBL-EBI search and sequence analysis tools APIs in 2019. Nucleic Acids Res..

[27] Lu S (2020). CDD/SPARCLE: The conserved domain database in 2020. Nucleic Acids Res..

[28] Mistry J (2021). Pfam: The protein families database in 2021. Nucleic Acids Res..

[29] Kelley LA, Mezulis S, Yates CM, Wass MN, Sternberg MJE (2015). The Phyre2 web portal for protein modeling, prediction and analysis. Nat. Protoc..

[30] Jumper J (2021). Highly accurate protein structure prediction with AlphaFold. Nature.

[31] Varadi M (2021). AlphaFold Protein Structure Database: Massively expanding the structural coverage of protein-sequence space with high-accuracy models. Nucleic Acids Res..

[32] Schmittgen TD, Livak KJ (2008). Analyzing real-time PCR data by the comparative *C*_T_ method. Nat. Protoc..

[33] Lacks SA, López P, Greenberg B, Espinosa M (1986). Identification and analysis of genes for tetracycline resistance and replication functions in the broad-host-range plasmid pLS1. J. Mol. Biol..

[34] Ruiz-Cruz S, Solano-Collado V, Espinosa M, Bravo A (2010). Novel plasmid-based genetic tools for the study of promoters and terminators in *Streptococcus pneumoniae* and *Enterococcus faecalis*. J. Microbiol. Methods.

[35] Brosius J, Dull TJ, Sleeter DD, Noller HF (1981). Gene organization and primary structure of a ribosomal RNA operon from *Escherichia coli*. J. Mol. Biol..

[36] Yanisch-Perron C, Vieira J, Messing J (1985). Improved M13 phage cloning vectors and host strains: Nucleotide sequences of the M13mp18 and pUC19 vectors. Gene.

[37] Qiu, Y., Zhai, C., Chen, L., Liu, X. & Yeo, J. Current insights on the diverse structures and functions in bacterial collagen-like proteins. *ACS Biomater. Sci. Eng.* 1c00018 (2021). 10.1021/acsbiomaterials.1c00018 33871954

[38] Lukomski S, Bachert BA, Squeglia F, Berisio R (2017). Collagen-like proteins of pathogenic streptococci. Mol. Microbiol..

[39] Rasmussen M, Edén A, Björck L (2000). SclA, a novel collagen-like surface protein of *Streptococcus pyogenes*. Infect. Immun..

[40] Almengor AC, McIver KS (2004). Transcriptional activation of *sclA* by Mga requires a distal binding site in *Streptococcus pyogenes*. J. Bacteriol..

[41] Almengor AC, Walters MS, McIver KS (2006). Mga is sufficient to activate transcription in vitro of *sof-sfbX* and other Mga-regulated virulence genes in the group A *Streptococcus*. J. Bacteriol..

[42] Humtsoe JO (2005). A streptococcal collagen-like protein interacts with the α_2_ β_1_ integrin and induces intracellular signaling. J. Biol. Chem..

[43] Caswell CC, Lukomska E, Seo N-S, Höök M, Lukomski S (2007). Scl1-dependent internalization of group A *Streptococcus* via direct interactions with the α_2_ β_1_ integrin enhances pathogen survival and re-emergence. Mol. Microbiol..

[44] Caswell CC, Oliver-Kozup H, Han R, Lukomska E, Lukomski S (2010). Scl1, the multifunctional adhesin of group A *Streptococcus*, selectively binds cellular fibronectin and laminin, and mediates pathogen internalization by human cells. FEMS Microbiol. Lett..

[45] Martín-Galiano AJ, García E (2021). *Streptococcus pneumoniae*: A plethora of temperate bacteriophages with a role in host genome rearrangement. Front. Cell. Infect. Microbiol..

[46] Muzzi A, Donati C (2011). Population genetics and evolution of the pan-genome of *Streptococcus pneumoniae*. Int. J. Med. Microbiol..

[47] Altschul SF (1997). Gapped BLAST and PSI-BLAST: A new generation of protein database search programs. Nucleic Acids Res..

[48] Aprianto R, Slager J, Holsappel S, Veening J-W (2018). High-resolution analysis of the pneumococcal transcriptome under a wide range of infection-relevant conditions. Nucleic Acids Res..

[49] Solano-Collado V (2021). Recognition of streptococcal promoters by the pneumococcal SigA protein. Front. Mol. Biosci..

[50] Ruiz-Cruz S, Moreno-Blanco A, Espinosa M, Bravo A (2018). DNA-binding properties of MafR, a global regulator of *Enterococcus faecalis*. FEBS Lett..

[51] Ruiz-Cruz S, Moreno-Blanco A, Espinosa M, Bravo A (2019). Transcriptional activation by MafR, a global regulator of *Enterococcus faecalis*. Sci. Rep..

[52] Studier FW, Moffatt BA (1986). Use of bacteriophage T7 RNA polymerase to direct selective high-level expression of cloned genes. J. Mol. Biol..

[53] Lacks SA (1966). Integration efficiency and genetic recombination in pneumococcal transformation. Genetics.

[54] Dower WJ, Miller JF, Ragsdale CW (1988). High efficiency transformation of *E*. *coli* by high voltage electroporation. Nucleic Acids Res..

