# Supplementary material for: PclR is a transcriptional activator of the gene that encodes the pneumococcal collagen-like protein PclA
Source: Sci Rep. 2022 Jul 12;12:11827. doi: 10.1038/s41598-022-15758-7 (PMC9276737; doi:10.1038/s41598-022-15758-7)
Supplement: Supplementary file 1 — Supplementary Information. [file 41598_2022_15758_MOESM1_ESM.pdf]

SUPPLEMENTARY MATERIAL

PclR is a transcriptional activator of the gene that encodes the pneumococcal collagen-like protein PclA

Ana Moreno-Blanco[1], Virtu Solano-Collado[1,2], Alejandro Ortuno-Camuñas[1], Manuel Espinosa[1], Sofía Ruiz-Cruz[1,3,]* and Alicia Bravo[1,]*

[1] Centro de Investigaciones Biológicas Margarita Salas, Consejo Superior de Investigaciones Científicas, Ramiro de Maeztu 9**,** E-28040 Madrid**,** Spain

[2] Present address: Institute of Medical Sciences, University of Aberdeen, Foresterhill. AB252ZD, Aberdeen, United Kingdom

[3] Present address: School of Microbiology, University College Cork & APC Microbiome Ireland, Western Road, Cork T12 YT20, Ireland

*****Corresponding authors
E-mail addresses: sofia.ruizcruz@ucc.ie (Sofía Ruiz-Cruz); abravo@cib.csic.es (Alicia Bravo)

Running title: PclR is a transcriptional activator

```
PclR      MRNLLSTKVQRQLRLMETLIQNRNWMKLHELAEKLGCTERILKSDLNELR 50
          ||:|||.|..|||.|:|.|.:::.|....||||.|.||||.:|.||:.::
MgaSpn    MRDLLSKKSHRQLELLELLFEHKRWFHRSELAELLNCTERAVKDDLSHVK 50

PclR      IAFPSINIQSSVNGIMIDLEVNTSVEDIYQYFLANSQSFQLLEYMFFNEG 100
          .|||.:...||.|||.|....::.:|.:|.:|..:|..|.:||::|||||
MgaSpn    SAFPDLIFHSSTNGIRIINTDDSDIEMVYHHFFKHSTHFSILEFIFFNEG 100

PclR      LPIYRTIENLYFSSANLYRLGRNITKVLSSQFQIELSFTPSEIRGNEIDI 150
          .......:..|.||::|||:...|.||:..|||.|:|.||.:|.|||.||
MgaSpn    CQAESICKEFYISSSSLYRIISQINKVIKRQFQFEVSLTPVQIIGNERDI 150

PclR      RYFFAQYFSERYYFLDWPFPDLPEEDLTEFADFFYKITNYPMRFSIYRMY 200
          ||||||||||:||||:|||.:...|.|::..:..||.|::||..|.:||.
MgaSpn    RYFFAQYFSEKYYFLEWPFENFSSEPLSQLLELVYKETSFPMNLSTHRML 200

PclR      KLMIAISIHRVKNGHFIDL-PNHFYKEYYPLLKSIPNFQETLAYFSKHFG 249
          ||::..:::|:|.|||::: .:.|..:....|......:.....|...:.
MgaSpn    KLLLVTNLYRIKFGHFMEVDKDSFNDQSLDFLMQAEGIEGVAQSFESEYN 250

PclR      LEMTPDTIAQIFISFLQNDIFLDPQEFFNSLEDNSQARYSYQLLSQILEG 299
          :.:..:.:.|:|:|:.|...|:|...|...::.:|....||.|||..::.
MgaSpn    ISLDEEVVCQLFVSYFQKMFFIDESLFMKCVKKDSYVEKSYHLLSDFIDQ 300

PclR      LSKQYKITFTNHDELIWHLHNTAFFERQEIFSTPILFEQKALTIKKFEVY 349
          :|.:|:|...|.|.|||||||||...|||:|:..|||:||..||:.|:..
MgaSpn    ISVKYQIEIENKDNLIWHLHNTAHLYRQELFTEFILFDQKGNTIRNFQNI 350

PclR      FPDFMGSARQELAQYRQAIGQHDHPEQLEHLMYTILTHAENLSTQLLENR 399
          ||.|:...::||:.|.:.:........:.||.||.:||.::|...||:|:
MgaSpn    FPKFVSDVKKELSHYLETLEVCSSSMMVNHLSYTFITHTKHLVINLLQNQ 400

PclR      PPIKVLIISNFDHAISLTFVDMLSYYCNNRFTFDIWDELKTSPEILNQTD 449
          |.:|||::||||...:....:.|||||:|.|..::|.||:.|.|.|..:.
MgaSpn    PKLKVLVMSNFDQYHAKFVAETLSYYCSNNFELEVWTELELSKESLEDSP 450

PclR      YDIIVSNFYIPGI-TKKFICRNHLSIMNLVNHLNTLSNEIHLSNTL 494
          ||||:|||.||.| .|:.|..|:::.::|:..||.:. .|.|..
MgaSpn    YDIIISNFIIPPIENKRLIYSNNINTVSLIYLLNAMM-FIRLDE-- 493
```

Figure S1. Alignment of PclR and Mga*Spn* according to EMBOSS Needle Pairwise Sequence Alignment (Rice *et al.*, 2000; Madeira *et al.*, 2019). The lines (|) indicate identical amino acid residues. The Protein Families Database (Pfam) (Mistry *et al.*, 2021) predicts that both proteins have two N-terminal DNA-binding domains: HTH_Mga (in blue, PF08280.14) and Mga (in red, PF05043.16).

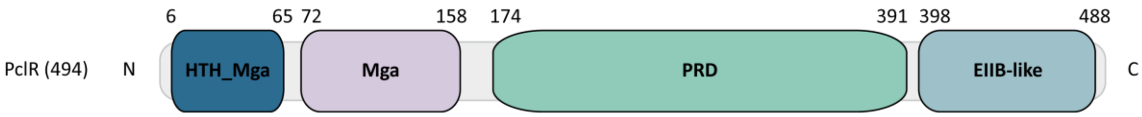

Figure S2. Organization of predicted functional domains in PclR (494 residues). PclR is predicted to have two N-terminal helix-turn-helix DNA-binding domains (HTH_Mga and Mga), a central PTS regulation domain (PRD), and a C-terminal region with structural homology to a PTS EIIB-like component (Lu *et al.*, 2020; Mistry *et al.*, 2021; Kelley *et al.*, 2015). The Mga*Spn* transcriptional regulator (493 residues) was predicted to have the same organization of functional domains (Solano-Collado *et al.*, 2012; Solano-Collado *et al.*, 2016).

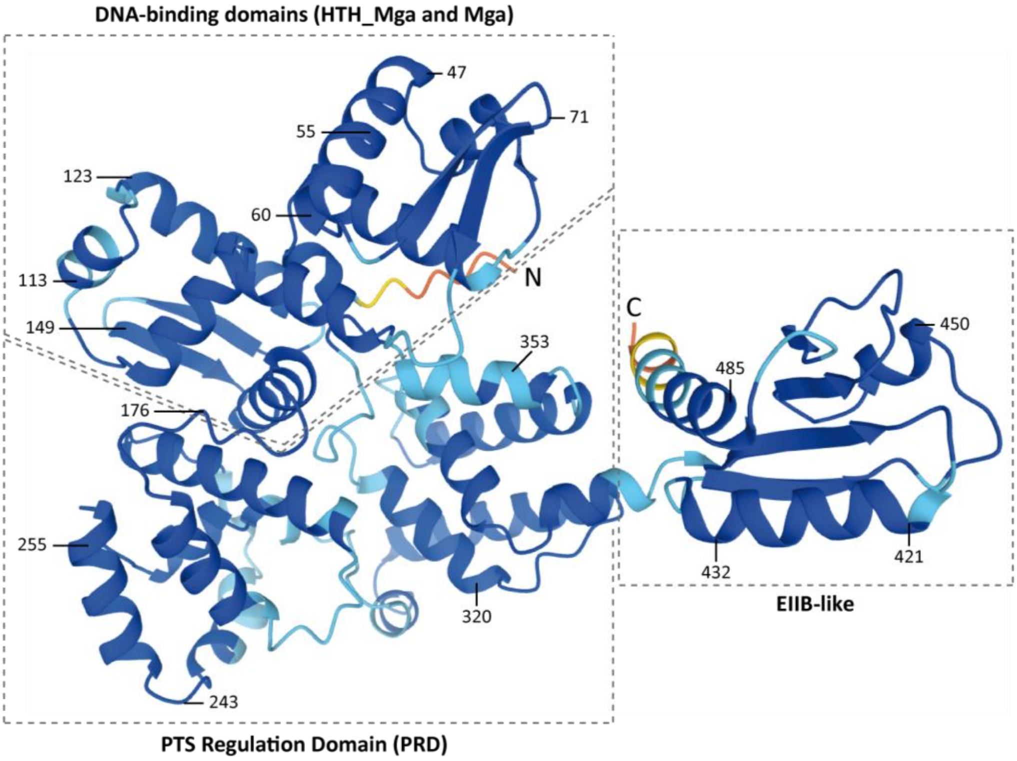

Figure S3. Predicted three-dimensional structure of the PclR monomer according to the AlphaFold Protein Structure Database (AlphaFold DB, https://alphafold.ebi.ac.uk) (Jumper *et al*., 2021; Varadi *et al*., 2021). The structure is coloured by the per-residue pLDDT confidence measure. Deep blue: very high confidence (pLDDT > 90). Light blue: confident (90 > pLDDT > 70). In the AlphaFold Database, the sequence of PclR (Spr1404) (https://alphafold.ebi.ac.uk/entry/Q8DP24) shows eight extra N-terminal residues preceding the initial Met residue (in orange, very low confidence, pLDDT < 50). The position of several residues is indicated as a reference. The regions that contain the predicted functional domains (see Figure S2) are shown.

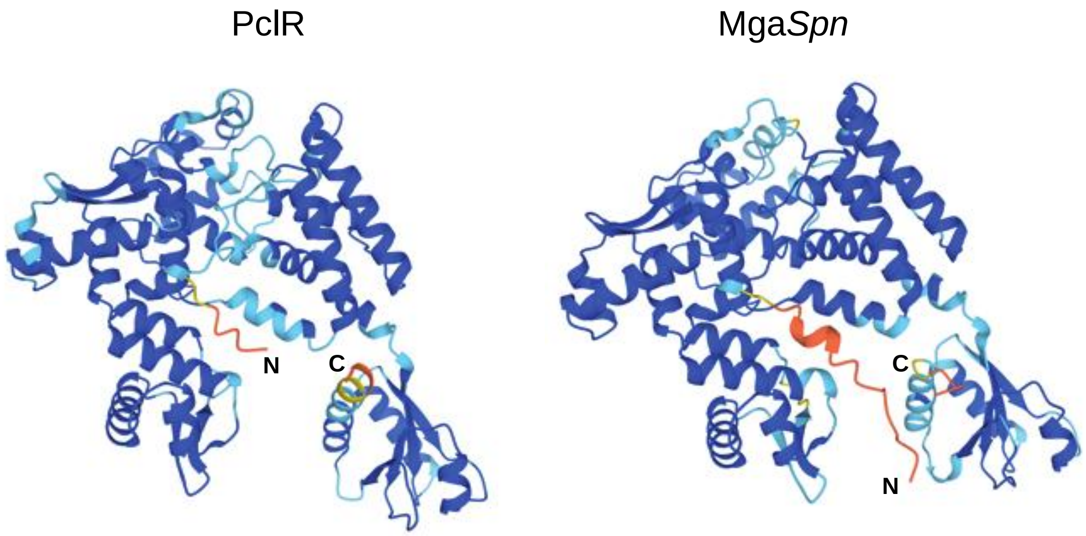

Figure S4. Predicted three-dimensional structures of the PclR and Mga*Spn* monomers according to the AlphaFold Protein Structure Database (AlphaFold DB, https://alphafold.ebi.ac.uk) (Jumper *et al*., 2021; Varadi *et al*., 2021). The structures are coloured by the per-residue pLDDT confidence measure. Deep blue: very high confidence (pLDDT > 90). Light blue: confident (90 > pLDDT > 70). In the AlphaFold Database, the sequence of PclR (Spr1404) (https://alphafold.ebi.ac.uk/entry/Q8DP24) shows eight extra N-terminal residues preceding the initial Met residue (in orange, very low confidence, pLDDT < 50), whereas the sequence of Mga*Spn* (Spr1622) (https://alphafold.ebi.ac.uk/entry/Q8DNN5) shows 19 extra N-terminal residues (in orange).

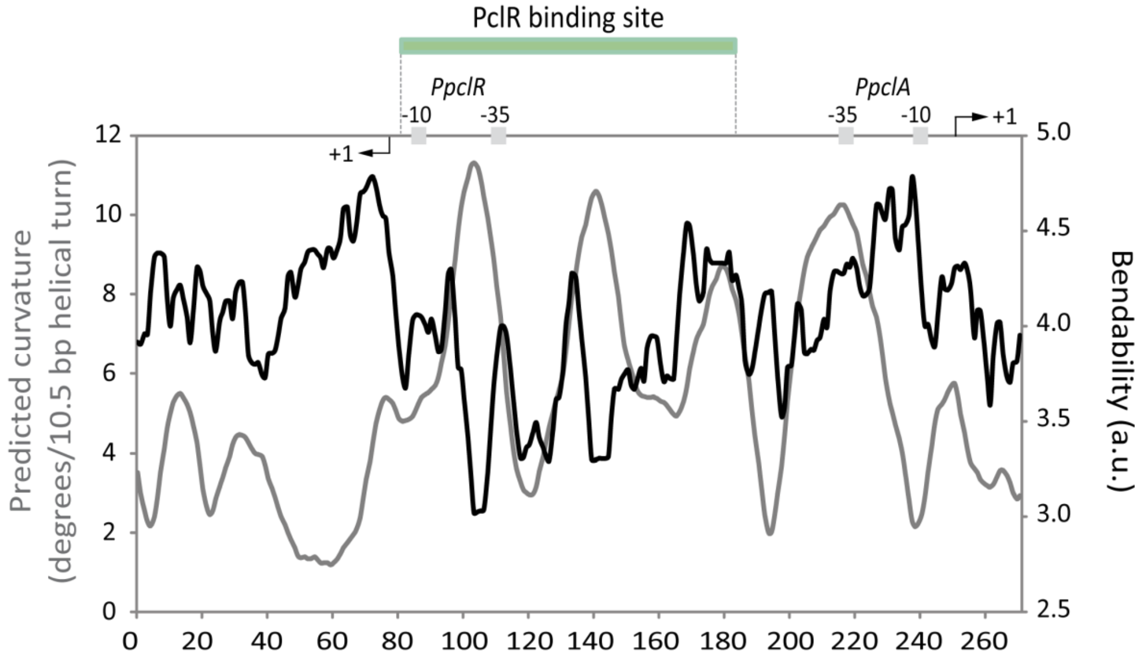

Figure S5. Bendability/curvature propensity plot of the 270-bp DNA fragment (coordinates 1388196-1387927 of the pneumococcal R6 genome) according to the bend.it server (pongor.itk.ppke.hu/dna/bend_it.html). The location of the *PpclR* and *PpclA* promoters, the transcription start site (+1 position) of the *pclR* and *pclA* genes, and the PclR binding site are indicated.

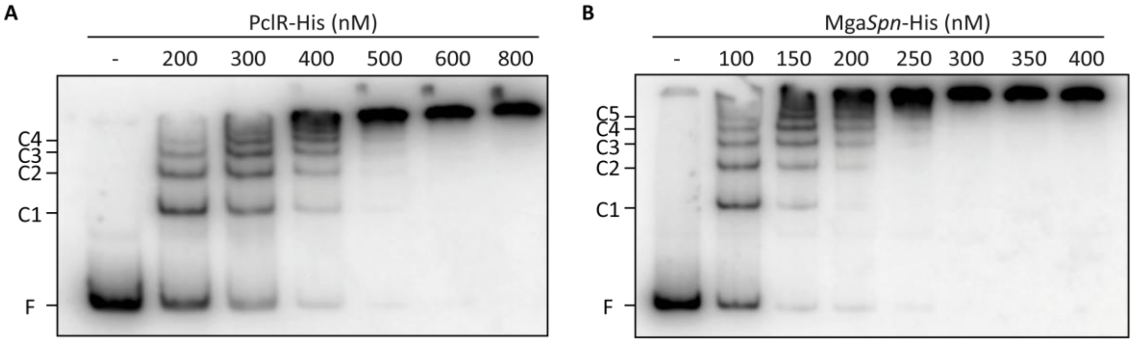

Figure S6. EMSA experiments. (A) Binding of PclR-His to the *PpclA* promoter region in the presence of competitor DNA (calf thymus DNA; 2 g/ml). (B) Binding of Mga*Spn*-His to the *PpclA* promoter region. In both experiments, the ^{32}P-labelled 270-bp DNA fragment (2 nM) (coordinates 1388196 to 1387927 of the R6 genome) was incubated with the indicated concentrations of protein. Free and bound DNAs were separated by native polyacrylamide (6%) gel electrophoresis. Labelled DNA was visualized using a Fujifilm Image Analyzer (FLA-3000). Bands corresponding to free DNA (F) and several protein-DNA complexes (C1 to C5) are indicated. **See the full-length gels at the end of the Supplementary Information file.**

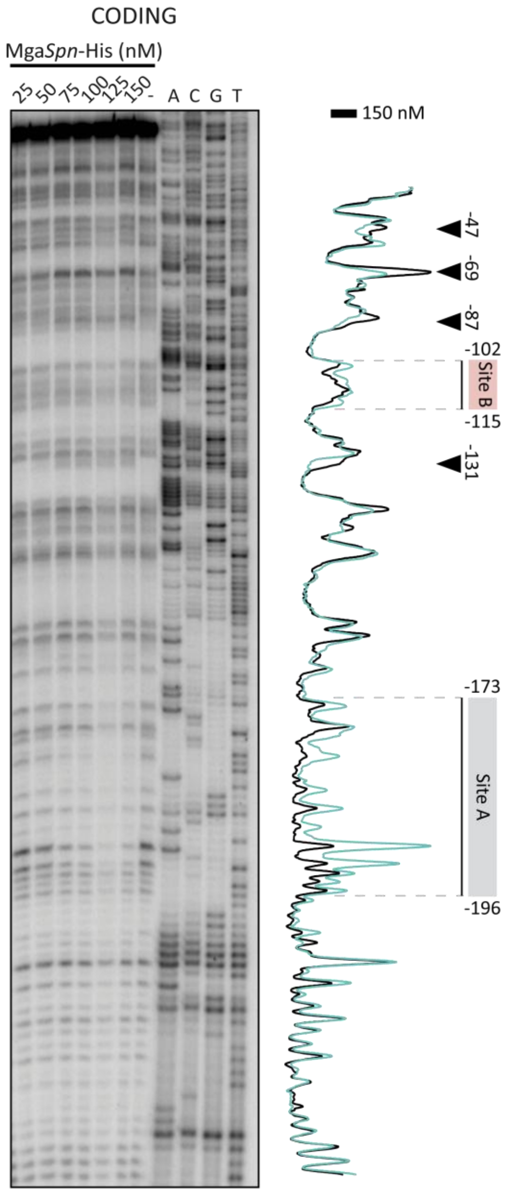

Figure S7. Sites recognized by Mga*Spn*-His on the *PpclA* promoter region as detected by DNase I footprinting. The 270-bp DNA fragment was ^{32}P-labelled at the 5´-end of the coding strand (relative to *pclA*) using the ^{32}P-labelled Dw*1404* oligonucleotide. Labelled DNA (2 nM) was incubated with the indicated concentrations of Mga*Spn*-His. Dideoxy-mediated chain termination sequencing reactions were run in the same gel (lanes A, C, G, T). The sequence corresponds to the coding strand of the 270-bp DNA fragment (^{32}P-labelled Dw*1404* oligonucleotide). Densitometer scans corresponding to DNA without Mga*Spn*-His (blue line) and DNA with Mga*Spn*-His (black line) are shown. Brackets represent the Mga*Spn*-His protected regions. Positions more sensitive to DNase I cleavage are indicated with arrowheads. The indicated positions are relative to the transcription start site (+1 position) of the *pclA* gene.

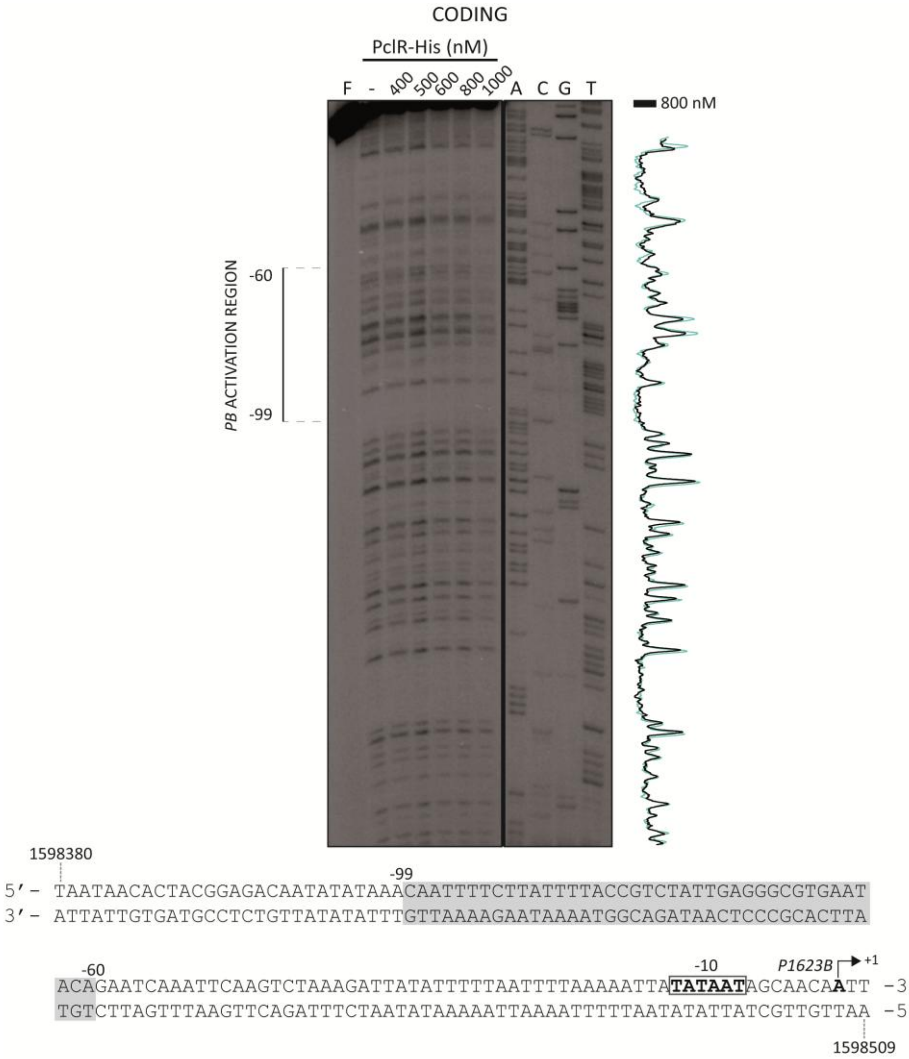

Figure S8. DNase I footprinting assay using PclR-His and the 222-bp DNA fragment (coordinates 1598298 to 1598519 of the R6 genome; see Solano-Collado *et al*., 2013). The DNA fragment was ^{32}P-labelled at the 5´-end of the coding strand (relative to the *P1623B* promoter) using the ^{32}P-labelled *1622*H oligonucleotide. The labelled DNA (2 nM) was incubated with the indicated concentrations of PclR-His. Non-digested DNA (F) and dideoxy-mediated chain termination sequencing reactions were run in the same gel (lanes A, C, G, T). The sequence corresponds to the coding strand of the 222-bp DNA fragment (^{32}P-labelled *1622*H oligonucleotide). Densitometer scans corresponding to DNA without PclR-His (blue line) and DNA with PclR-His (black line) are shown. All the lanes came from the same gel (delineation with dividing lines). The nucleotide sequence of the region spanning coordinates 1598380 to 1598509 is shown. The transcription initiation site (+1 position) of the *spr1623* gene and the -10 element of the *P1623B* promoter are indicated. The *PB* activation region (from -60 to -99) that contains the site recognized by the Mga*Spn* regulator (Solano-Collado *et al*., 2013) is indicated with a bracket on the left of the gel.

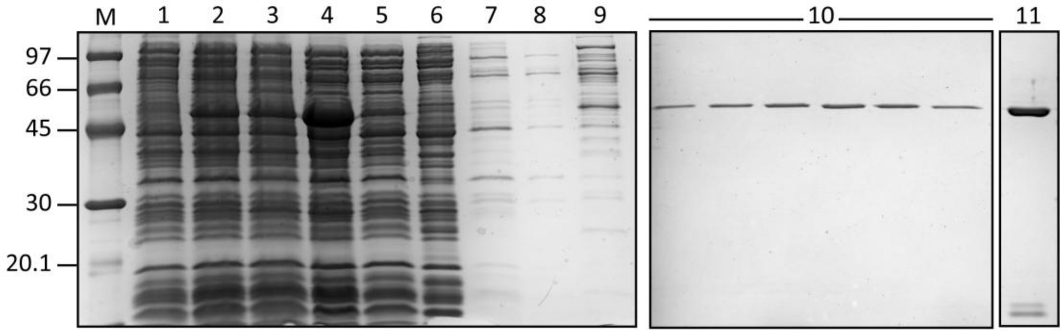

Figure S9. Purification of PclR-His. Basically, we used the protocol reported previously for MafR-His (Ruiz-Cruz *et al.*, 2018). Protein fractions were analysed by SDS-polyacrylamide (12%) gel electrophoresis. Gels were stained with Coomassie Blue. Lane M: molecular weight standards (in kDa) were run in the same gel (LMW Marker, GE Healthcare). Induction of *pclR*-His gene expression: whole-cell extract before adding IPTG (lane 1), whole-cell extract after treatment with IPTG for 25 min (lane 2), whole-cell extract after treatment with IPTG for 25 min, and then with rifampicin for 60 min (lane 3). Purification steps: whole-cell extract after using a French Pressure Cell Disruptor (lane 4), cleared cell lysate (lane 5), supernatant after precipitation of nucleic acids with PEI (0.2%) in the presence of 300 mM NaCl (lane 6), proteins eluted from the PEI pellet using the same buffer with 300 mM NaCl (lanes 7-8), proteins eluted from the PEI pellet using a higher ionic strength buffer (700 mM NaCl) (lane 9), PclR-His eluted from the nickel affinity column using a linear gradient of imidazole (10-250 mM) (lanes 10), and preparation of PclR-His after concentration (lane 11). **See the full-length gels at the end of the Supplementary Information file.**

Table S1: Relative expression of the *pclR* gene in strain R6 at the logarithmic (R6-Log) and stationary (R6-Sta) growth phases

Strain	cDNA	C_T *pclR* (1)	C_T *era* (2)	$2^{-\Delta CT}$ (3)	Mean ± sd (4)	FC(5)	p-value
R6-Log	1	23.44	19.69	0.074			
	2	23.18	19.69	0.089	0.080 ± 0.007		
	3	23.72	20.03	0.077			
						3.20	0.042
R6-Sta	1	24.60	20.03	0.042			
	2	25.96	20.25	0.019	0.025 ± 0.014		
	3	26.33	20.19	0.014			

Table S2: Relative expression of the *mgaSpn* gene in strain R6 at the logarithmic (R6-Log) and stationary (R6-Sta) growth phases

Strain	cDNA	C_T *mgaSpn*(1)	C_T *era* (2)	$2^{-\Delta CT}$ (3)	Mean ± sd (4)	FC(5)	p-value
R6-Log	1	21.79	19.69	0.234			
	2	22.08	19.69	0.190	0.218 ± 0.024		
	3	22.14	20.03	0.230			
						4.36	0.028
R6-Sta	1	23.11	20.03	0.118			
	2	26.04	20.25	0.018	0.050 ± 0.058		
	3	26.23	20.19	0.015			

R6 cells were grown under standard laboratory conditions to an optical density at 650 nm of 0.2 (logarithmic phase) and 0.9 (stationary phase). For each growth phase, total RNA was isolated from three independent bacterial cultures. cDNA (random primers) was synthesized from each RNA preparation. From each cDNA sample, three PCRs per gene were performed.

(1) Mean C_T from the PCRs for *pclR* (Table S1) and *mgaSpn* (Table S2), respectively
(2) Mean C_T from the PCRs for the *era* gene (internal control gene)
(3) ΔC_T = C_T gene under study - C_T internal control gene
(4) Mean ± standard deviation of the $2^{-\Delta CT}$ values
(5) Fold change in expression of the studied gene

See graphical representation in Supplementary Figure S10

Table S3: Relative expression of the *pclR* gene in R6 harbouring plasmid pDLF*pclR* (strain A) and R6 harbouring plasmid pDLF*pclR-i* (strain B)

Strain	cDNA	C_T *pclR* (1)	C_T *era* (2)	$2^{-\Delta CT}$ (3)	Mean ± sd (4)	FC(5)	p-value
	1	19.99	20.58	1.508			
A	2	19.68	20.25	1.485	1.368 ± 0.222		
	3	19.46	19.61	1.111			
						3.12	0.047
	1	21.92	20.08	0.279			
B	2	21.54	20.39	0.451	0.438 ± 0.153		
	3	20.81	20.04	0.585			

Table S4: Relative expression of the *pclR* gene in R6Δ*mga* harbouring plasmid pDLF*pclR* (strain C) and R6Δ*mga* harbouring plasmid pDLF*pclR-i* (strain D)

Strain	cDNA	C_T *pclR* (1)	C_T *era* (2)	$2^{-\Delta CT}$ (3)	Mean ± sd (4)	FC(5)	p-value
	1	21.92	22.71	1.734			
C	2	19.93	20.77	1.785	1.823 ± 0.113		
	3	19.98	20.94	1.950			
						4.94	0.007
	1	23.18	22.20	0.505			
D	2	22.69	20.96	0.301	0.369 ± 0.118		
	3	22.82	21.09	0.301			

For each strain, total RNA was isolated from three independent bacterial cultures. cDNA (random primers) was synthesized from each RNA preparation. From each cDNA sample, three PCRs per gene were performed.

(1) Mean C_T from the PCRs for the *pclR* gene

(2) Mean C_T from the PCRs for the *era* gene (internal control gene)

(3) $\Delta C_T = C_T$ *pclR* gene - C_T *era* gene

(4) Mean ± standard deviation of the $2^{-\Delta CT}$ values

(5) Fold change in expression of the *pclR* gene

See graphical representation in Supplementary Figure S10

Table S5: Relative expression of the *pclA* gene in R6 harbouring plasmid pDLF*pclR* (strain A) and R6 harbouring plasmid pDLF*pclR-i* (strain B)

Strain	cDNA	C_T *pclA* (1)	C_T *era* (2)	$2^{-\Delta CT}$ (3)	Mean ± sd (4)	FC(5)	p-value
	1	22.46	19.87	0.166			
A	2	22.94	20.06	0.136	0.146 ± 0.018		
	3	23.32	20.44	0.136			
						3.39	0.009
	1	24.61	20.13	0.045			
B	2	24.73	20.38	0.049	0.043 ± 0.007		
	3	25.14	20.28	0.034			

Table S6: Relative expression of the *pclA* gene in R6Δ*mga* harbouring plasmid pDLF*pclR* (strain C) and R6Δ*mga* harbouring plasmid pDLF*pclR-i* (strain D)

Strain	cDNA	C_T *pclA* (1)	C_T *era* (2)	$2^{-\Delta CT}$ (3)	Mean ± sd (4)	FC(5)	p-value
	1	23.22	20.93	0.204			
C	2	20.55	18.20	0.197	0.195 ± 0.011		
	3	21.23	18.78	0.183			
						4.53	0.0015
	1	22.68	18.06	0.041			
D	2	23.51	19.25	0.052	0.043 ± 0.008		
	3	23.21	18.44	0.037			

For each strain, total RNA was isolated from three independent bacterial cultures. cDNA (random primers) was synthesized from each RNA preparation. From each cDNA sample, three PCRs per gene were performed.

(1) Mean C_T from the PCRs for the *pclA* gene

(2) Mean C_T from the PCRs for the *era* gene (internal control gene)

(3) $\Delta C_T = C_T$ *pclA* gene - C_T *era* gene

(4) Mean ± standard deviation of the $2^{-\Delta CT}$ values

(5) Fold change in expression of the *pclA* gene

See graphical representation in Supplementary Figure S10

Table S7: Relative expression of the *pclA* gene in R6Δ*mga* harbouring plasmid pDL*PsulA::mga* (strain E) and R6Δ*mga* harbouring plasmid pDL287 (strain F)

Strain	cDNA	C_T *pclA* (1)	C_T *era* (2)	$2^{-\Delta CT}$ (3)	Mean ± sd (4)	FC(5)	p-value
	1	23.28	20.20	0.118			
E	2	23.50	19.16	0.049	0.081± 0.034		
	3	23.49	19.74	0.074			
						1.39	0.26
	1	23.61	19.70	0.066			
F	2	23.95	19.35	0.041	0.058 ± 0.014		
	3	23.50	19.58	0.066			

For each strain, total RNA was isolated from three independent bacterial cultures. cDNA (random primers) was synthesized from each RNA preparation. From each cDNA sample, three PCRs per gene were performed.

(1) Mean C_T from the PCRs for the *pclA* gene
(2) Mean C_T from the PCRs for the *era* gene (internal control gene)
(3) ΔC_T = C_T *pclA* gene - C_T *era* gene
(4) Mean ± standard deviation of the $2^{-\Delta CT}$ values
(5) Fold change in expression of the *pclA* gene

See graphical representation in Supplementary Figure S10

Table S8: Relative expression of the *spr1623* gene in R6 harbouring plasmid pDLF*pclR* (strain A) and R6 harbouring plasmid pDLF*pclR-i* (strain B)

Strain	cDNA	C_T *1623* (1)	C_T *era* (2)	$2^{-\Delta CT}$ (3)	Mean ± sd (4)	FC(5)	p-value
	1	16.68	19.87	9.149			
A	2	16.59	20.06	11.074	11.191 ± 2.103		
	3	16.70	20.44	13.350			
						1.12	0.12
	1	17.03	20.13	8.545			
B	2	17.05	20.38	10.091	9.927 ± 1.307		
	3	16.80	20.28	11.145			

Table S9: Relative expression of the *spr1623* gene in R6Δ*mga* harbouring plasmid pDLF*pclR* (strain C) and R6Δ*mga* harbouring plasmid pDLF*pclR-i* (strain D)

Strain	cDNA	C_T *1623* (1)	C_T *era* (2)	$2^{-\Delta CT}$ (3)	Mean ± sd (4)	FC(5)	p-value
	1	19.55	21.71	4.485			
C	2	18.72	19.88	2.242	3.073 ± 1.229		
	3	18.62	19.93	2.492			
						1.16	0.39
	1	19.13	20.85	3.294			
D	2	19.16	20.20	2.067	2.648 ± 0.615		
	3	18.62	19.99	2.582			

For each strain, total RNA was isolated from three independent bacterial cultures. cDNA (random primers) was synthesized from each RNA preparation. From each cDNA sample, three PCRs per gene were performed.

(1) Mean C_T from the PCRs for the *spr1623* gene
(2) Mean C_T from the PCRs for the *era* gene (internal control gene)
(3) $\Delta C_T = C_T$ *spr1623* gene - C_T *era* gene
(4) Mean ± standard deviation of the $2^{-\Delta CT}$ values
(5) Fold change in expression of the *spr1623* gene

See graphical representation in Supplementary Figure S10

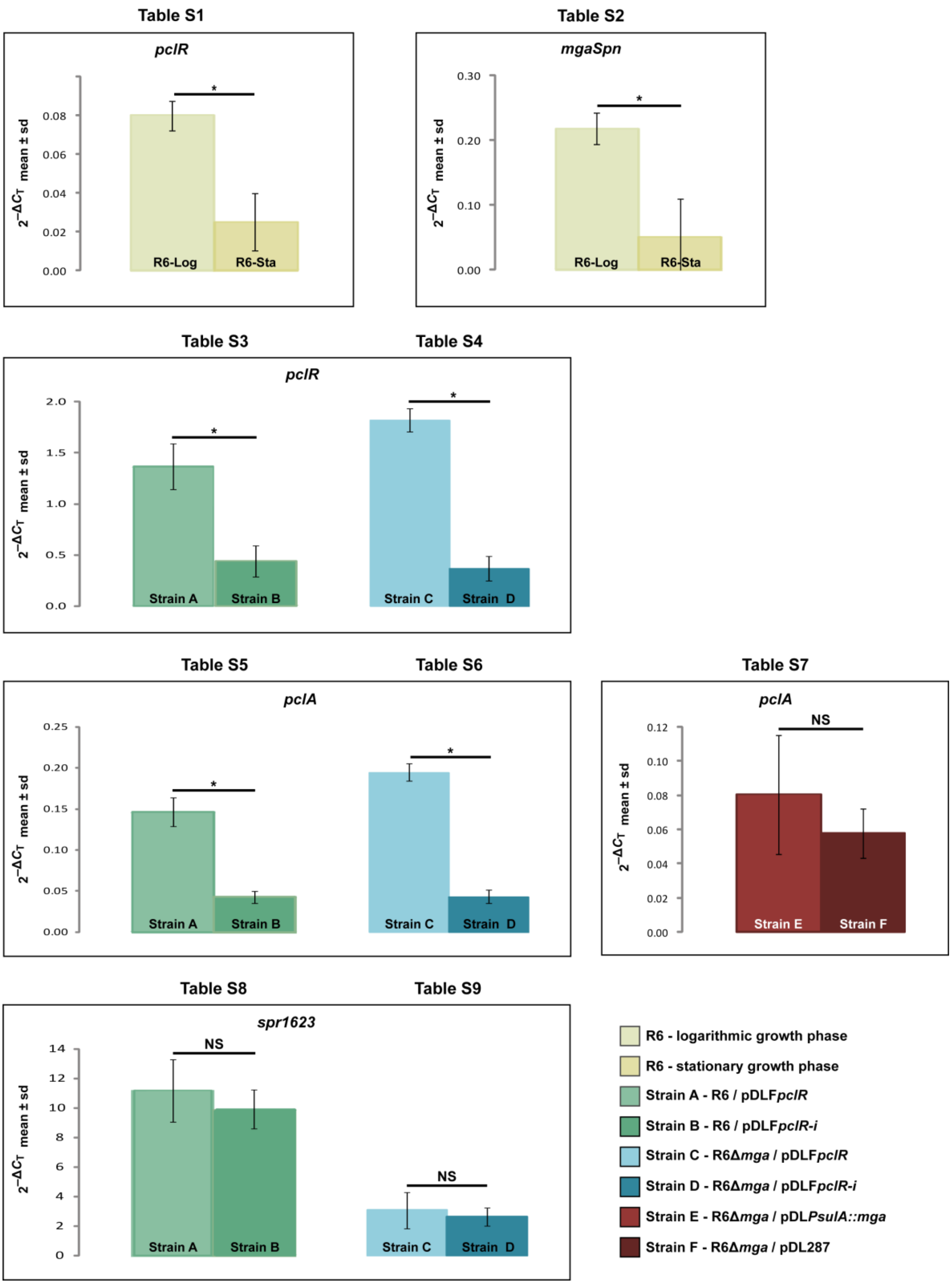

Figure S10. Graphical representation of the results from Tables S1 to S9 (mean ± standard deviation of the $2^{-\Delta CT}$ values). P-values were calculated using the Student´s *t*-test (paired, two-tailed); *p < 0.05, NS, Non-significant.

Table S10. Presence of the *pclR* gene in some pneumococcal genomes

Pneumococcal genomes	PclR (Identity with PclR from R6)
AP200	NO
ATCC 700669	YES (99.60%)
A026	YES (99.39%)
CGSP14	NO
D39	YES (100%)
gamPNI0373	NO
G54	NO
Hungary 19A-6	NO
INV104	YES (99.39%)
INV200	NO
JJA	YES (99.60%)
OXC141	NO
P1031	NO
SPNA45	NO
SPN034156	NO
SPN034183	NO
SPN994038	NO
SPN994039	NO
ST556	YES (99.39%)
Taiwan19F-14	YES (99.39%)
TCH8431/19A	YES (99.39%)
TIGR4	NO
670-6B	NO
70585	YES (99.80%)

We selected 24 pneumococcal strains whose genomes are totally sequenced. To know whether these genomes encoded PclR, we used the BLASTP protein sequence alignment program (Altschul *et al*., 1997). As a query, we used the sequence of PclR from strain R6 (494 amino acids; NCBI Reference Sequence WP_001245194.1).

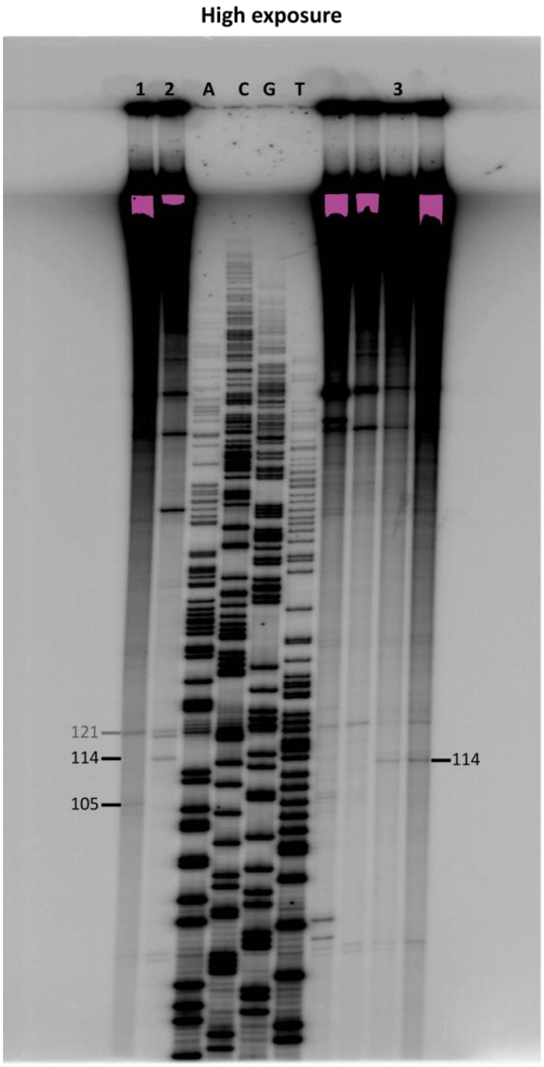

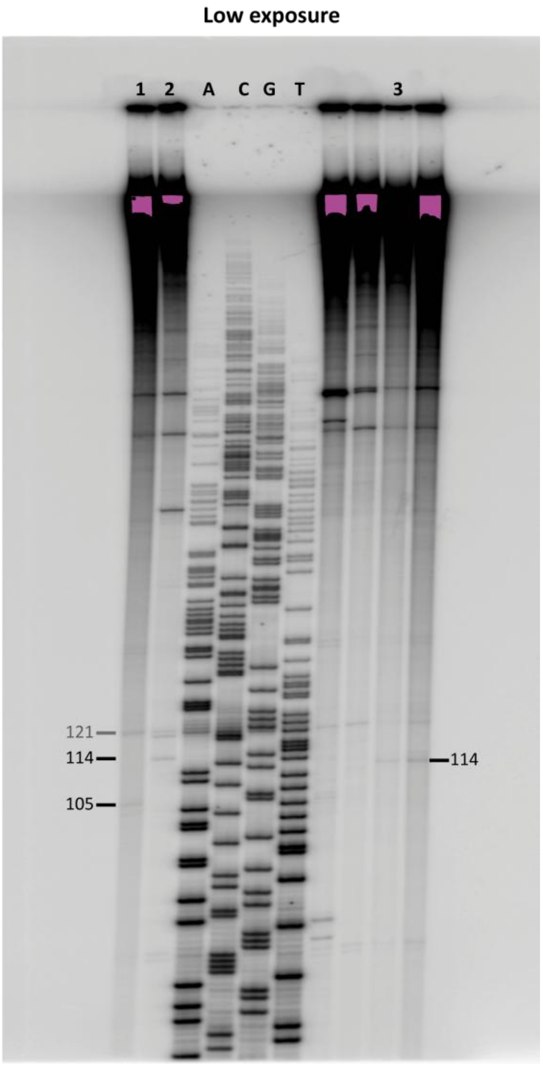

Supplementary Originals of the Main Manuscript Figures 2C and 3C (primer extension reactions). The full-length gel used to compose panels C of Figures 2 and 3 is shown. High and low exposures of the same full-length gel are shown. Lanes 1 and 2 in the full-length gel (high exposure) correspond to lanes 1 and 2 of Figure 2C, respectively. Lane 3 in the full-length gel (high exposure) corresponds to lane 1 of Figure 3C. Lanes A, C, G, and T of the full-length gel (low exposure) were used to compose Figures 2C and 3C. The dideoxy-sequencing reactions (lanes A, C, G, and T) were run in the same gel just as DNA size markers. See the legends in Figures 2 and 3 for details.

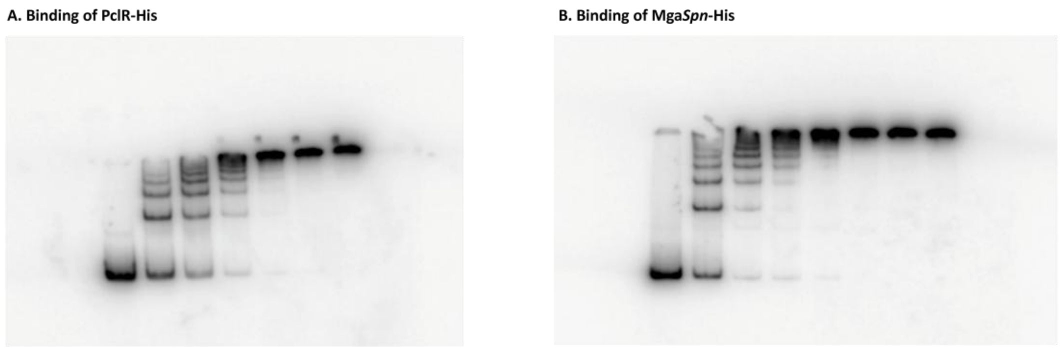

Supplementary Originals of the Supplementary Figure S6 (EMSA experiments). The full-length gels used to compose panels A and B of Figure S6 are shown. See the legend in Figure S6 for details.

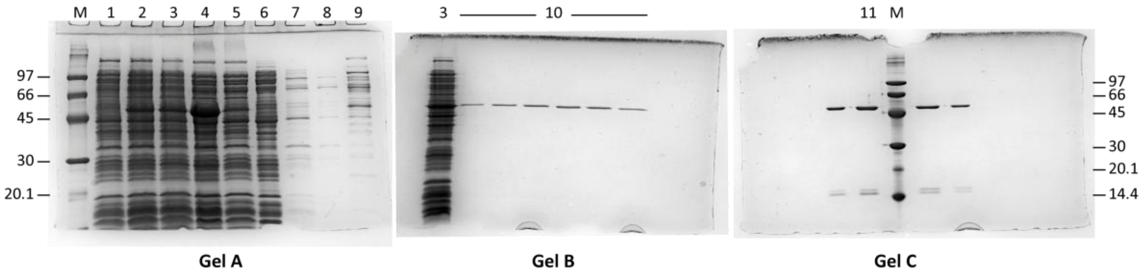

Supplementary Originals of the Supplementary Figure S9 (purification of PclR-His). The full-length gels used to compose Figure S9 are shown. Lane M and lanes 1 to 9 in gel A correspond to lane M and lanes 1 to 9 of Figure S9, respectively. Lanes denoted 10 in gel B correspond to lanes denoted 10 in Figure S9. Lane 11 in gel C corresponds to lane 11 of Figure S9. See the legend in Figure S9 for details.
